# A life cycle assessment of disposing intra-operative collected fluids, a comparative study between the Neptune 3 versus canister drainage

**DOI:** 10.1038/s41598-025-20375-1

**Published:** 2025-10-21

**Authors:** L. Droog, J. van der Sijp, T. Hoppe, J. Huegel, L. Tariq, T. Horeman, Bart van Straten

**Affiliations:** 1 Leiden University & Delft University of Technology, Industrial Ecology, Leiden, The Netherlands; 2 Haaglanden Medical Center, The Hague, The Netherlands; 3 Delft University of Technology, Delft, The Netherlands; 4 OLVG Hospital, Amsterdam, The Netherlands; 5 GreenCycl Field Lab, De Meern, Utrecht, The Netherlands; 6 Stryker Instruments, Portage, MI USA; 7 Stryker European Regional Headquarters, Amsterdam, The Netherlands; 8 Department: BioMechincal Engineering Institution, Delft University of Technology, Mekelweg 2, Delft, The Netherlands

**Keywords:** Life cycle assessment, Climate change, Sustainable, Circular economy, Neptune 3, Canisters, Health care economics, Health policy, Biomedical engineering, Mechanical engineering, Ecological modelling

## Abstract

The Neptune 3 drainage system is developed as an alternative to canister systems for collecting surgical fluids in the operating room. This study investigates the difference in the environmental impact between Neptune 3 and canister systems, evaluating all individual stages of the product life cycle of the Neptune and canisters. Using the RECIPE model, 17 impact categories (midpoints) were defined and results were aggregated into three endpoint categories (human health, ecosystems, resources). The volume of waste was varied in a setup in a hospital in the Netherlands and included different fluid volume collection scenarios performed over seven years: high-volume (2.0–24 L waste) and low-volume (0.1–0.5 L waste). In both high and low volume procedures, Neptune 3 has a lower environmental impact compared to canisters for global warming (15–89% reduction), ozone formation terrestrial ecosystems & human health (24–91% reduction), fossil resource scarcity (36–92% reduction) and water consumption (44–106% reduction). In high volume scenarios (5 + Liters) Neptune also has a lower impact in stratospheric ozone depletion, fine particulate matter formation, terrestrial acidification for the high volume scenarios (5 L or more). In the case of ionizing radiation, freshwater eutrophication, and human carcinogenic toxicity the Neptune has a lower impact only in the very large volume procedures (10 + Liter). By aggregating the mid-point results to end-point results, it is observed that the Neptune system is beneficial for resources in each scenario, and for human health and ecosystems for procedures with larger volumes. Results from this LCA demonstrate that the Neptune 3 system is environmentally beneficial compared to canisters. This study provides valuable information for policymakers and hospital decisionmakers to treat their surgical waste in an environmentally friendly way.

**Supplementary Information:**

The online version contains supplementary material available at 10.1038/s41598-025-20375-1.

## Introduction

Hospitals have a significant environmental footprint; one contributor is the production of both hazardous and non-hazardous waste^1^. The operating room (OR) creates a variety of waste types including stainless steel and plastic as well as fluids and gases used during surgical procedures; each of these require a waste disposal stream. In the United States, hospitals produce 5.9 million tons of waste annually and they are responsible for 8% of the country’s total CO_2_ emissions^2^. Similarly, the total CO_2_ emissions attributed to healthcare in the Netherlands are approximately 11 Mton, which represents 8% of the total CO_2_ footprint of this country^3,4^.

In recent years, the Dutch industrial and healthcare systems have become increasingly interested in the development of new technology, processes, and methods that reduce hospital waste and energy consumption to reduce operational costs^4^ and CO_2_ emissions. During the COVID-19 pandemic, the introduction of the European Green Deal and resulting programs on Sustainable Healthcare increased public awareness of climate change and intensified the interest in increasing hospital sustainability^4^. The Green Deal was established in 2019 and has since been signed by over 300 industrial and government organisations. With the mission to become a climate-neutral continent, one goal is to reduce healthcare CO_2_ emissions by up to 55% by 2030 (when compared to emission levels in 1990)^4,5^.

The COVID-19 pandemic also promoted an interest in improved safety of OR staff when dealing with waste flow. Cross contamination between patients and staff during handling of contaminated materials can cause infections and have other hazardous health effects^6–12^.

The conventional method and current standard for many hospitals for collecting and storing surgical fluid waste is to suction fluid into plastic bag-lined canisters (referred to as a canister system)^13–15^. These plastic canisters are then disposed of via waste incineration. To address the management of fluid waste and smoke associated with surgical procedures, the Neptune 3 Waste Management System (Stryker, Kalamazoo, Michigan USA) was developed to collect, transport, and dispose of surgical fluid waste in the sewer (Fig. 2). The constantly closed nature of this system reduces contact with collected waste and could also reduce the risk of contamination due to exposure when compared to traditional canister waste collection systems^16^. This study investigates the difference in the environmental impact between the novel approach of the Neptune 3 and the traditional canister systems.

**Fig. 1:**
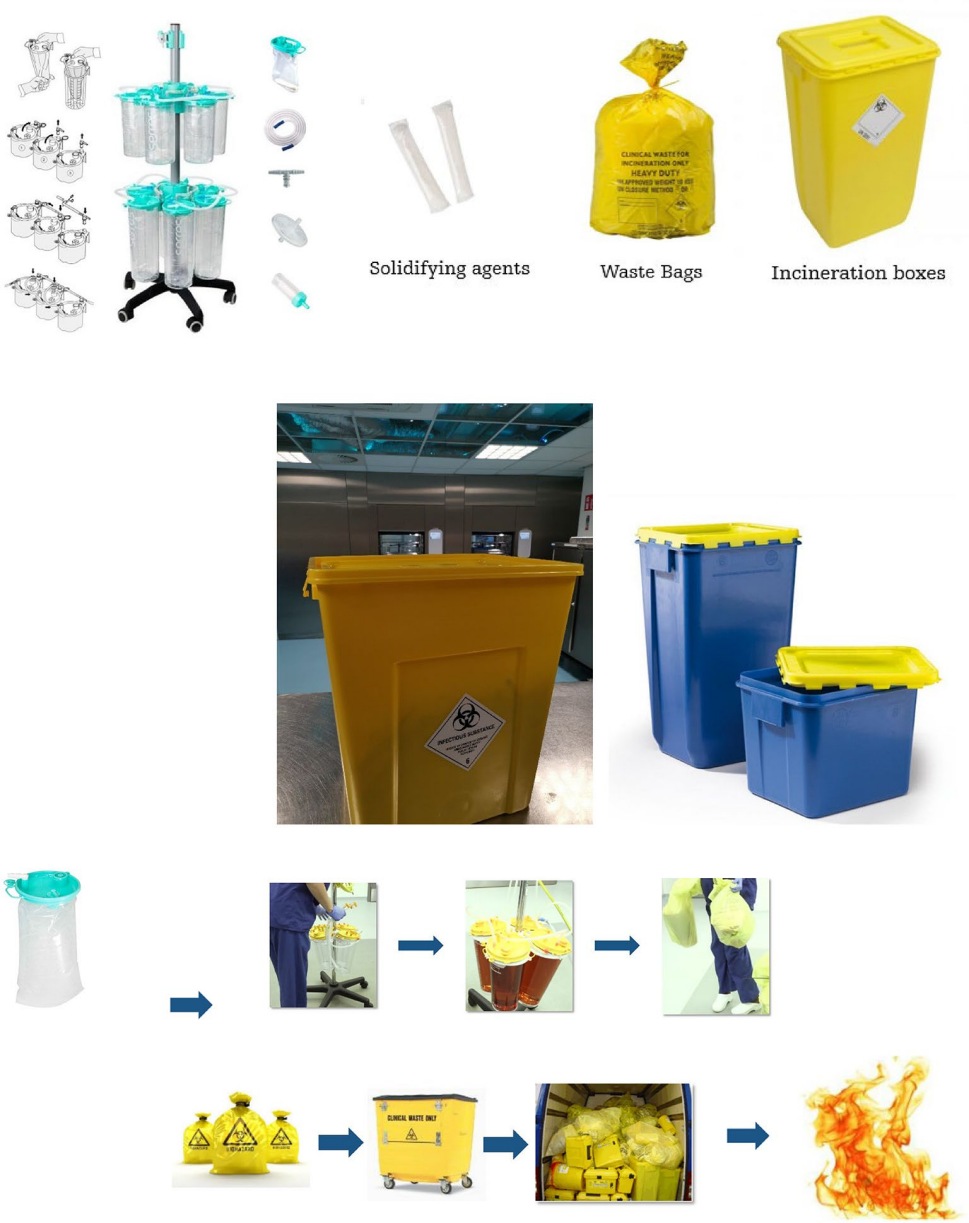
Typical waste treatment of surgical fluid waste with canisters and *WIVA *container with a click lid for transport of healthcare waste.

Typical waste treatment of surgical fluid waste (i.e. irrigation fluid) utilizes disposable collection products such as suction canisters^17^ and WIVA containers^18^ as shown in Fig. 1. During surgery, typically one staff member is responsible for managing fluid collection. Used plastic suction canisters and collected fluid waste are disposed of and transported to be incinerated at high temperatures in a special incineration installation designed to treat hazardous waste^19–21^. This scenario first requires hospital resources with regards to the storage, transport, and processing of fluid waste material. This waste collection and disposal method can also create unsafe situations or pose a health hazard as OR personnel must lift heavy fluid-filled containers^22^. This form of waste processing is also an energy intensive process due to the requirement to incinerate liquids. Burning of medical waste negatively impacts the environment due to associated CO_2_ emission, exhausting nitrous oxide, carcinogens including but not limited dioxins.; burning of fluids lowers the temperature of the incineration process and requires additional energy input to maintain the correct temperature, increasing the environmental impact^23^. Moreover, costly single-use collection containers have to be used for transportation which are incinerated^24,25^.

**Fig. 2:**
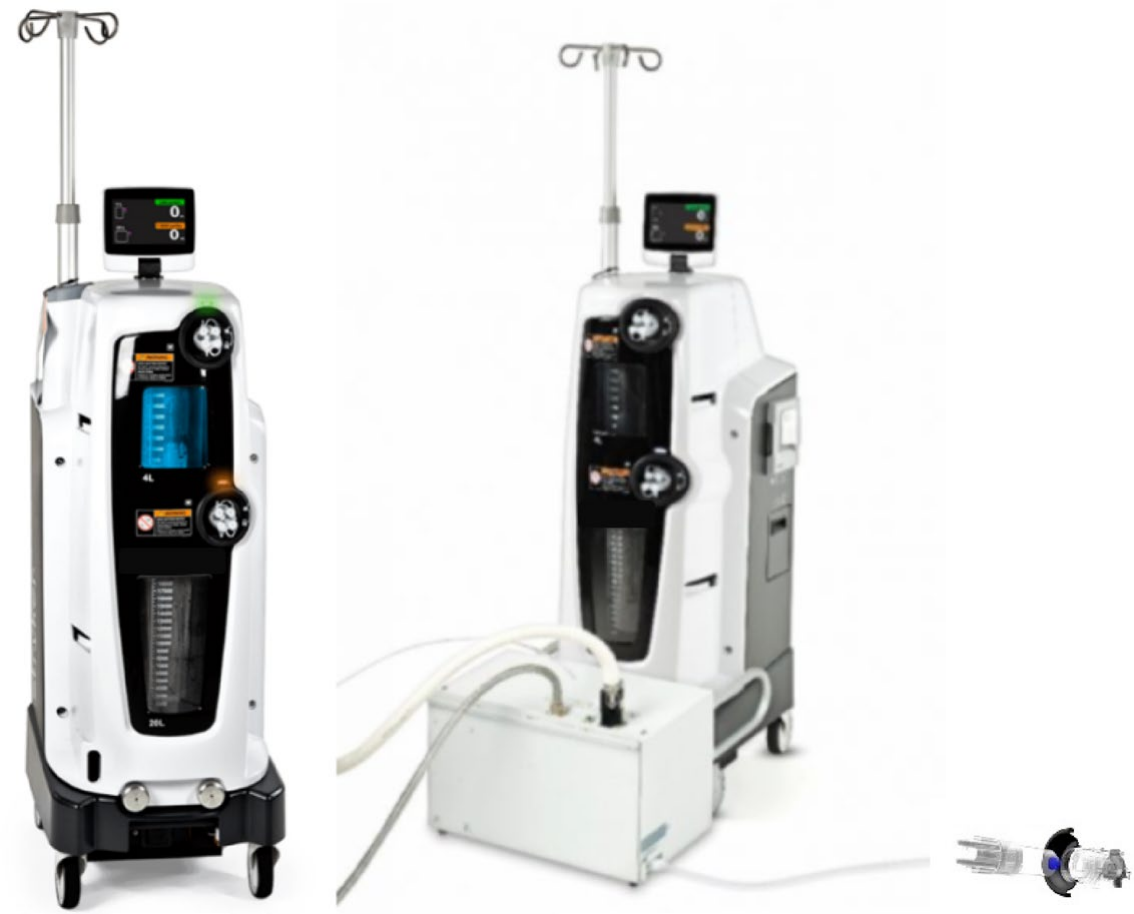
Neptune 3 waste management system, Rover module left and with components (right).

The Neptune 3 Waste Management System is a surgical suction system that is used to collect and dispose of fluid waste associated with surgical procedures. The Neptune 3 as shown in Fig. 2 is a closed mobile, closed system for fluid waste management; the constantly closed nature of this system protects the OR staff from exposure to suctioned fluids. Suction tubes remove blood to improve visibility of the surgical site in addition to collecting irrigation fluid and surgical smoke. The system consists of two appliances: the rover and the docking station. The mobile rover is present in the operating room and for each procedure, a disposable manifold provides a patient-specific fluid path from the suction lines to the rover collection container^26^. Via the docking station, fluids collected by the rover are automatically removed and the collection container is cleaned.

The docking station can be used by multiple rovers that are present in the operating room or hospital system. Fluids transferred from the rover to the docking station are disposed via direct connection to the hospital’s drainage system and therefore to the sewerage system. This procedure is in line with Central Sterilization and Services Departments (CSSD) of hospitals. In comparison, the departments where contaminated instruments are rinsed, washed, disinfect, sterilized also dispose dirty water on the sewerage system. Also, municipal wastewater treatment plants handle biological contaminants to an acceptable risk level and according to hospital infection control policies and local water discharge regulations. This is a common procedure in hospitals in many countries.

Although the hardware of the Neptune system is designed to last for more than ten years, for the purpose of this study, a depreciation time of seven years is assumed.

The objective of this study was to conduct a Life Cycle Assessment (LCA) to compare two processes of collecting and disposing of intra-operative fluid waste: Stryker’s Neptune 3 and conventional drainage by means of suction canisters. This comparison will determine whether the local disposal of surgical fluids from the OR using a device such as the Neptune 3 is more environmentally friendly than decentralized incineration. The LCA will assess and compare the environmental impact of local disposal of surgical fluids by means of a Neptune 3 versus the collection in suction canisters including the transportation and final disposal by incineration. Similar approaches have been reported in literature where the environmental impact of medical related products, such as face masks, were evaluated by means of the LCA method^27^. The LCA will compare the following mid-point categories: global warming, stratospheric ozone depletion, ionizing radiation, ozone formation (human health & terrestrial ecosystems), fine particulate matter formation, terrestrial acidification, freshwater eutrophication, marine eutrophication, terrestrial ecotoxicity, freshwater ecotoxicity, marine ecotoxicity, human carcinogenic toxicity, human non-carcinogenic toxicity, land use, mineral resource scarcity, fossil resource scarcity and water consumption.

## Methods

The environmental impact of two surgical fluid waste management systems, the Neptune 3 and traditional canisters, are evaluated using the life cycle assessment (LCA) ISO standards ISO 14,040 and 14,044 standards^28,29^. The individual stages of the product’s life cycle starting from raw material extraction to production, packaging, transport, use, reprocessing and final disposal of the Neptune 3 and suction canisters are evaluated^30^. The LCA was performed using SimaPro software^31^. Life cycle impact analysis data were retrieved from the “ecoinvent” database (Ecoinvent version 3.6, Zürich, Switzerland)^32^.

The LCA defines the ‘functional unit’ as performing the ‘primary function’ of the evaluated products; in this study, the primary function is the collection and disposal of intra-operative collected fluids. The functional unit^33^ is described in Supplementary Table S1 Functional Units, representing a range from 0.1 to 24 L of intra-operative collected fluids. A number of scenarios were assessed due to variation in surgical fluids used and collected in different procedural types. An overview defining this range via High- and Low-volume scenarios may be found in Supplementary Table S1 Functional Units. The resulting functional unit is defined as providing the collection and disposal of intra-operative fluid waste at these defined fluid volumes and case volumes over seven years of procedures.

Combining the two fluid waste collection systems and the defined functional units establishes two reference flows. Flow 1: The collection and disposal of intra-operative collected fluids over 7 years of procedures in high-volume and low-volume scenarios with the use of the Neptune 3 system and Flow 2: collection and disposal of intra-operative collected fluids over 7 years of procedures in high-volume and low-volume scenarios with the use of the canister system.

The following categories were defined as midpoint indicators for assessment in this study: global warming, stratospheric ozone depletion, ionizing radiation, ozone formation (human health & terrestrial ecosystems), fine particulate matter formation, terrestrial acidification, freshwater eutrophication, marine eutrophication, terrestrial ecotoxicity, freshwater ecotoxicity, marine ecotoxicity, human carcinogenic toxicity, human non-carcinogenic toxicity, land use, mineral resource scarcity, fossil resource scarcity and water consumption. Midpoint indicators are defined as an environmental mechanism relating to final measures of environmental impact as defined by endpoint indicators. Although the midpoints all effect the endpoints, it is to be assumed that the scale at which the waste collection systems are used (surgical case volume) will not impact the endpoints.

A Neptune 3 System was placed at OLVG Hospital in Amsterdam. The data collection form can be found in Supplementary File 1.

The results of the impact categories are determined by applying the ReCiPe midpoint characterization model.

Life cycle inventory (LCI) analysis.

System boundaries.

**Fig. 3:**
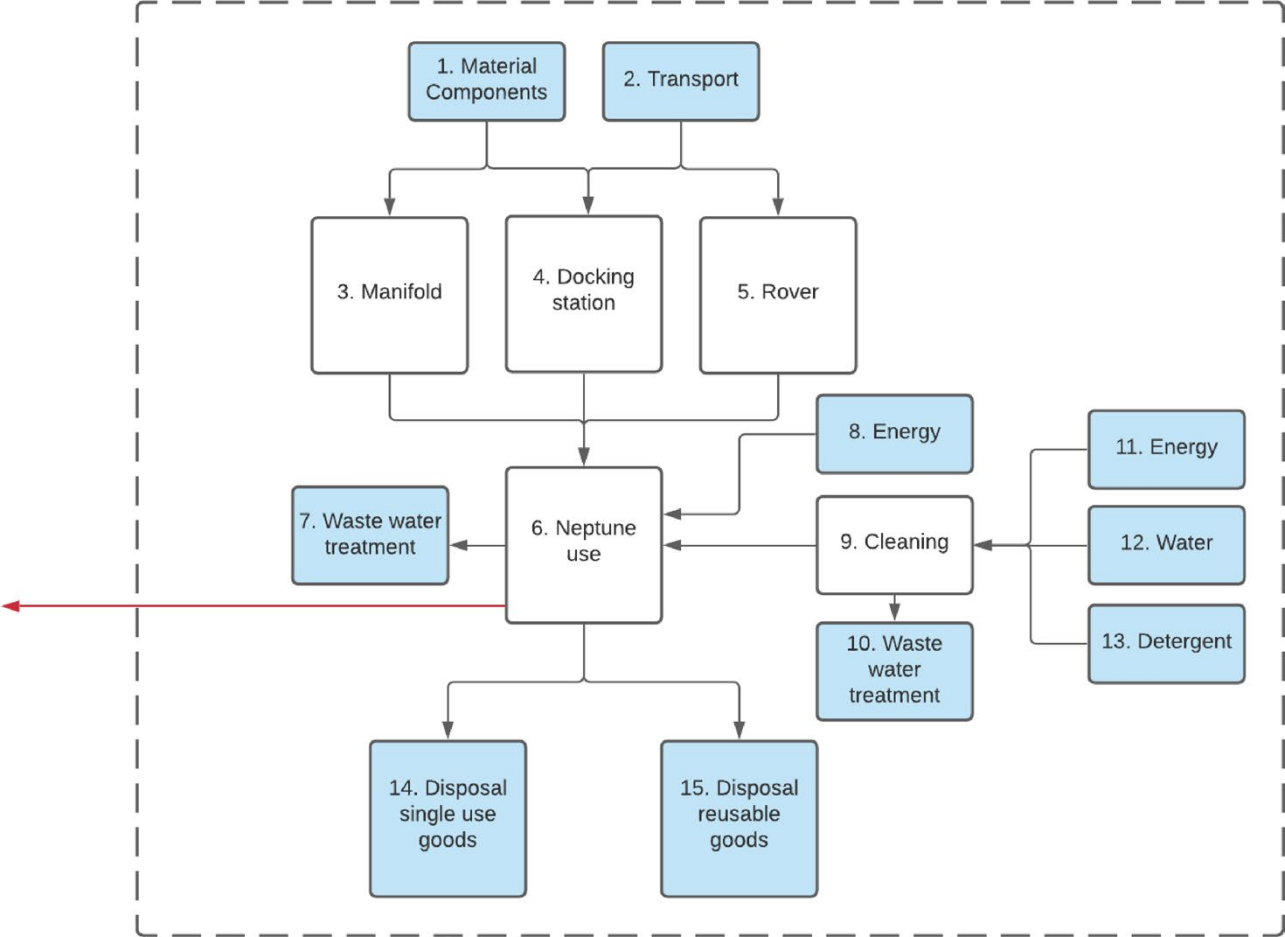
System boundaries Neptune 3 system depicted with the dotted line. The blue areas show the background process, the white areas represent the unit processes.

**Fig. 4:**
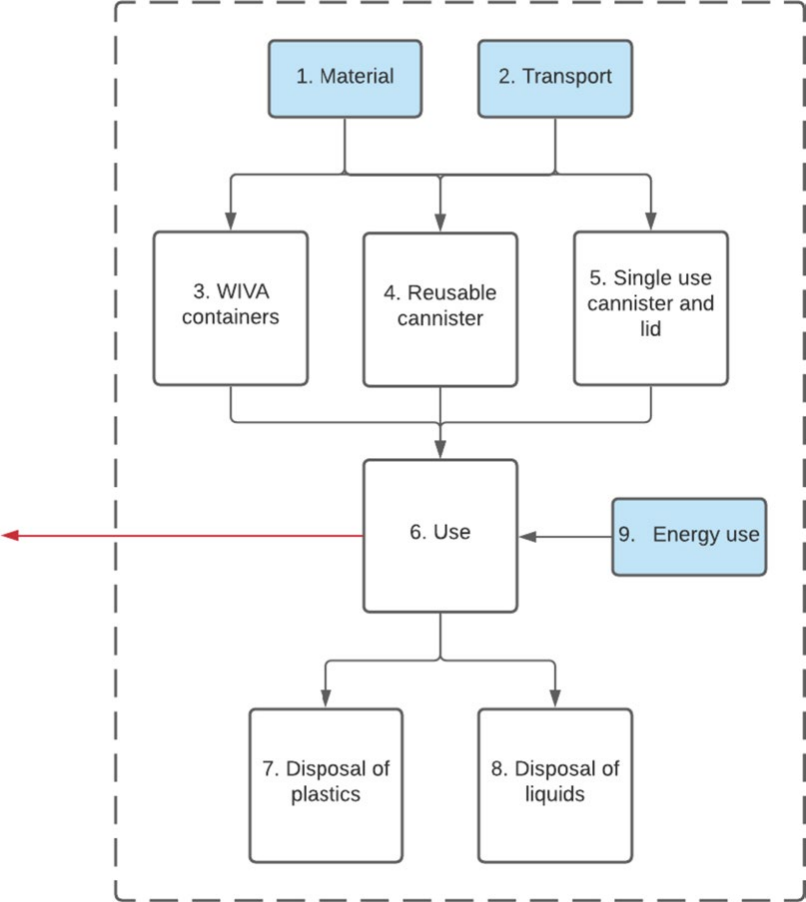
System boundaries conventional canisters depicted with the dotted line. The blue areas show the background process, the white represent the unit processes.

The study focuses on the collection and disposal of intra-operative fluid waste resulting from high- and low-volume procedures in the operating room. The life cycle encompasses all processes related to the production of the required equipment and appliances, the transport, the electricity, and water used related to the collection in the OR and the final disposal and treatment of the collected fluids as shown in the flowchart as depicted in Fig. 3 for Neptune 3 and Fig. 4 for the suction canister system. In these flowcharts, the blue boxes represent background processes, whereas white boxes represent foreground processes. Input for background processes are directly obtained from Ecoinvent, whereas the foreground processes are directly related to Neptune 3.

The flowchart of Neptune 3 starts with the production of the rover, docking station and manifolds. For each appliance, the main material and weight of the components is used to establish the environmental impact caused by the production of the device. Moreover, product transport from the location of the production of the components to final assembly as well as distribution route are included. Next, the use of Neptune is included in the system boundary. This encompasses requirements for electricity as well as water and detergent utilized in the Neptune cleaning process. Lastly, the environmental impact of product disposal and wastewater treatment are included.

The system boundaries for suction canisters are similar to the system boundaries of Neptune 3. Here, the flowchart starts with the material and transport related to the production of the required equipment. Hereafter, the collected fluids and used equipment is in its entirety transported for incineration.

### Economy-environment system boundary

In the case of both alternatives, the subtraction of raw/virgin materials results in the inclusion of different environmental flows in the background processes. Eventually, these extracted elements will leave the economic system again as environmental flows when the material is incinerated after use.

### Cut-off

The energy, buildings, machinery, and personnel related to the production, distribution and use of the appliances and equipment are disregarded from this study as this was not feasible to estimate with the current data availability on these processes. Moreover, the stand on which the canisters are placed during use are not included due to the long lifespan of this product. Finally, the vacuum system (e.g. material) used for the canisters is disregarded as its main purpose is related to the air conditioning of the OR. Nevertheless, the energy use related to the creation of vacuum required to use the canisters is included.

### Data collection

The material input of the suction canister was based on the Bemis, 1500 cc Liner (Bemis Suction Canister, Bemis Health Care, Sheboygan Falls, USA). Moreover, the production location of the material related to the use of canisters was assumed to be in Shanghai, China, as this is the most common origin from which to ship products from the manufacturing site to other continents^19^. Further distribution took place from Bracknell, UK to Neuss, Germany with the final destination set in Rotterdam, the Netherlands. The weight and material of 1050 g and Polypropylene, respectively, of the 30-liter WIVA-container is based on information provided by the supplier (Alertshop, Deurne, the Netherlands). The energy use of the canisters is based on the energy use of a mobile canister system of which information is provided by the supplier (Serres, n.d.)^34^. The use of cannisters per scenario is simulated and with the following assumptions: a minimum of one 2-litre suction canister and one 30-litre WIVA container are used, each procedure uses the suction system effectively for 20 min and all material including collected liquids are incinerated at Zavin, Dordrecht, the Netherlands.

Furthermore, the main material, weight, and location of production of each of the components used for the Neptune 3 are provided by the producer’s suppliers. Based on the location of the production of the components and the location of the final assembly and the distribution route the total amount of transport is calculated from the producer of the components to the producer of the Neptune 3 and suction canisters, to the distribution centres and final users.

The inventory data related to distribution and waste treatments are primarily based on background information obtained from ecoinvent 3.6. However, the waste treatment flows in ecoinvent are considered to be insufficient for the incineration of suction canisters due to the large quantity of fluids that require additional energy input for the gasification process.

Therefore, a new process was created specifically focused on incinerating fluids using the following in- flows and outflows:

- Economic input of municipal waste incineration facility: 2.5E-10 unit.

*Based on incineration processes in Ecoinvent*.

- Economic input of heat, natural gas: Q = m ∙ c ∙ ∆ T.

m = mass = 1 kg.

c = specific heat = 4186 J/kg∙ °C.

∆T = difference in temperature = 100–15 = 85 °C.

Q = heat energy = 0.36 MJ.

- Economic input of transport, lorry: 0.0911 TKM.

*Based on distance between OLVG Oost and Zavin = 91.1 KM and transport of 0.001-ton liquid waste*.

- Environmental outflow of heat equal to the input: 0.36 MJ.
- Environmental outflow of water equal to the input: 1 kg.

The energy usage of Neptune 3 is determined by measuring during use within the hospital environment. A linear regression model was created based on 34 measurements over procedures in which a wide range of surgical liquids were collected (0.3 to 40 L). The resulting model in the form E[y_i]= β_0 + β_1 x_i with a confidence level of 99% was as following: E[y_i] = 0.33 + 0.025x_i. In this case xi is the number of collected Liters during a procedure and E[yi] the expected value at xi.

This equation is illustrated using the 24-Liter scenario as an example, which resulted in:

(0.33 + 0.025 ∙ 24) ∙ 400 = 372 kWh.

These calculations resulted in the following values:

**Table:** 

Liters	Energy use per procedure (kWh)	number of procedures	Total kWh
24	0.93	400	372
20	0.83	400	332
10	0.58	400	232
7	0.505	400	202
5	0.455	800	364
2	0.38	800	304
0.5	0.3425	550	188.38
0.4	0.34	550	187
0.3	0.3375	550	185.63
0.2	0.335	550	184.25
0.1	0.3325	550	182.875

A full inventory analysis can be found in Supplementary Figure S1 Inventory Analysis which includes Fig. 2 (Inventory Cannisters) and different liter scenarios (Supplementary Tables S3 – S35).

### Life cycle impact assessment

The results of the inventory table are translated into contributions to relevant environmental impact categories to compare the two fluid waste management alternatives and to evaluate the product systems. This study initially considers the impact categories defined in the ReCiPe model. The results of the impact categories are determined by applying the ReCiPe midpoint characterization model. The midpoint results were converted into the endpoint categories of human health, ecosystems, and resources using the ReCiPe endpoint characterization model.

The environmental indicators from the inventory tables are assigned to the above-defined impact categories. The characterization factors from the ReCiPe characterization model are coupled to the environmental flows with the SimaPro software, through which the results are acquired.

## Results

All results are shown in Table 1. The parameters for which Neptune 3 is the better performing alternative are marked in green, red numbers indicate an inferior performance for Neptune, and yellow indicates differences that are not significant. Fluid waste system and procedure volume are noted in the left most column for each assessment.

**Table 1:**
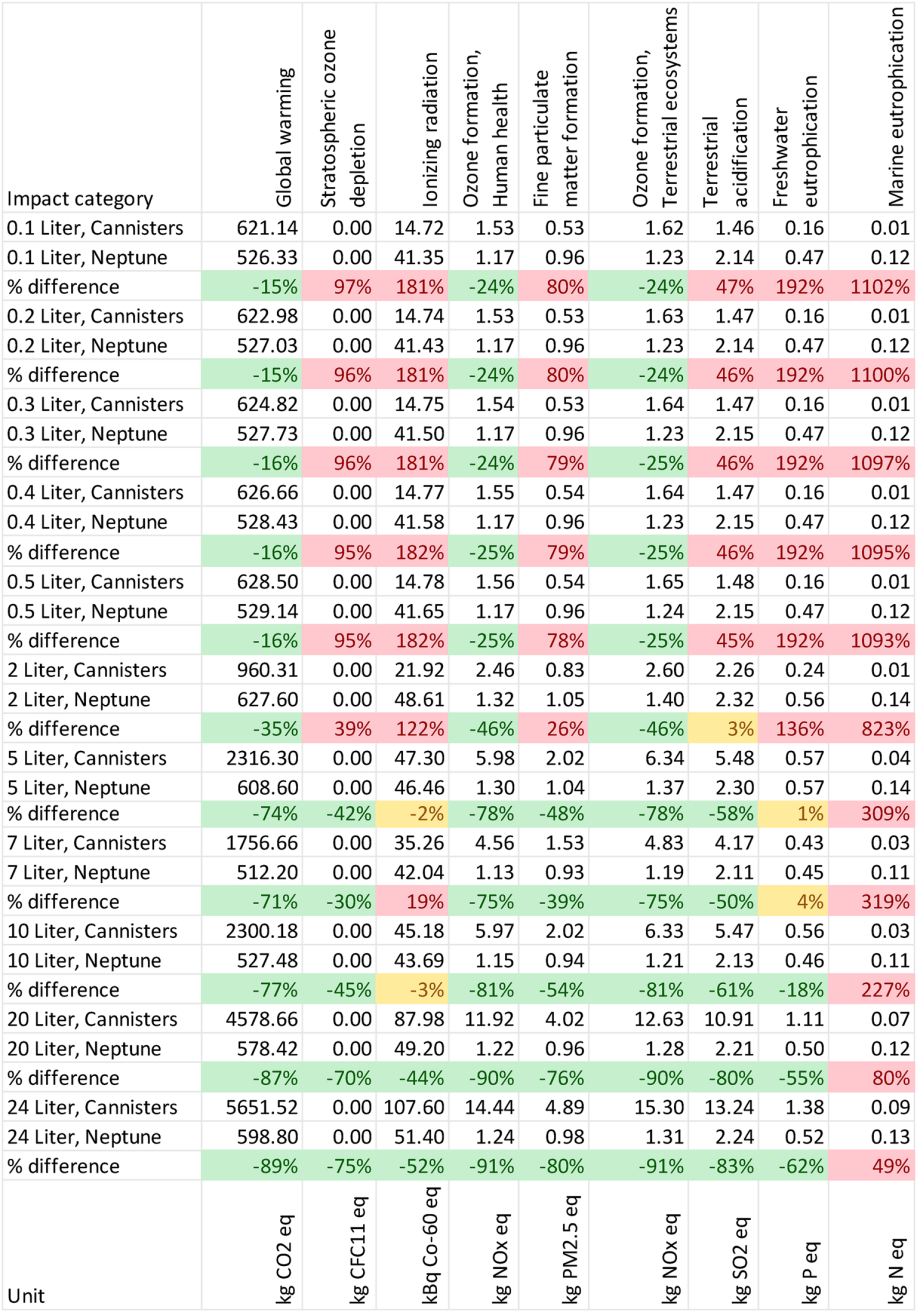

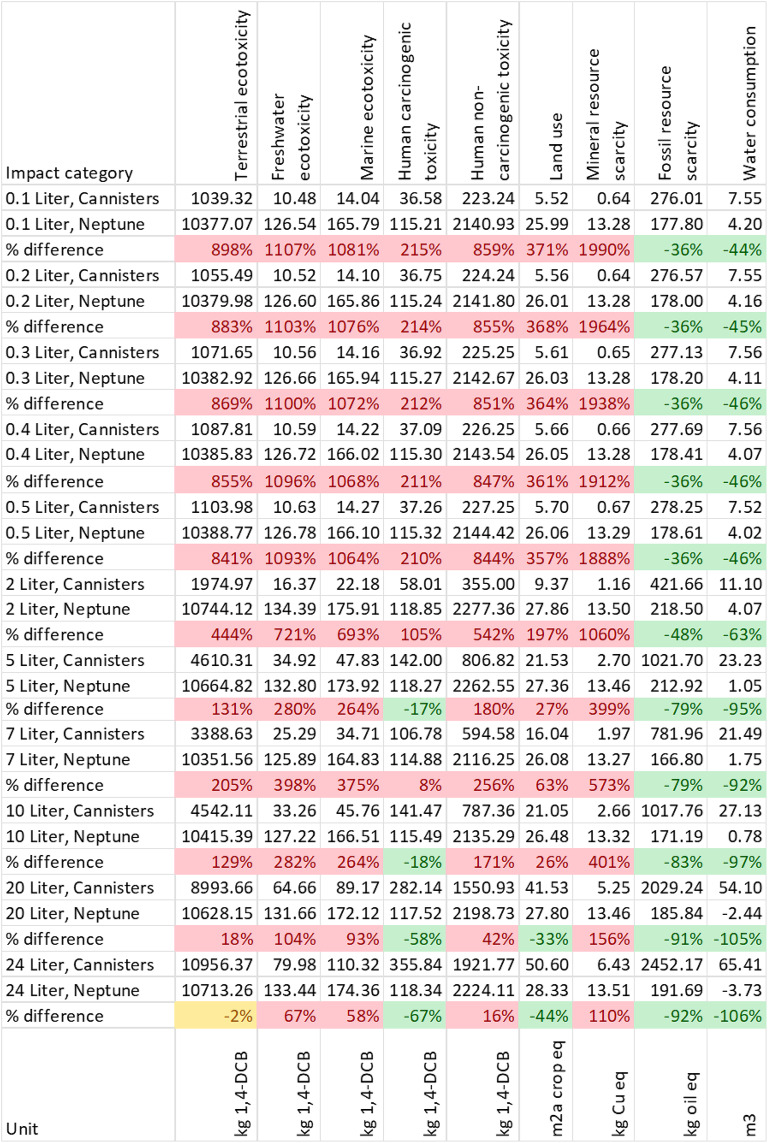
Midpoint results and relative difference for all impact categories.

The results show that Neptune 3 has a lower environmental impact across both low and high volume procedures for global warming (15–89% reduction), ozone formation terrestrial ecosystems & human health (24–91% reduction), fossil resource scarcity (36–92% reduction) and water consumption (44–106% reduction). The Neptune also has a lower impact in stratospheric ozone depletion, fine particulate matter formation, terrestrial acidification for procedures with volume of 5 L and more. In the case of Ionizing radiation, freshwater eutrophication, and human carcinogenic toxicity the Neptune only scores better in procedures with volume of 10 L and more. The Neptune has a larger impact for categories of marine eutrophication, terrestrial ecotoxicity, freshwater ecotoxicity, marine ecotoxicity, human non-carcinogenic toxicity, land use and mineral scarcity, with the difference between Neptune and canisters increasing as procedure volume decreases.

### Results on the endpoint categories

**Fig. 5:**
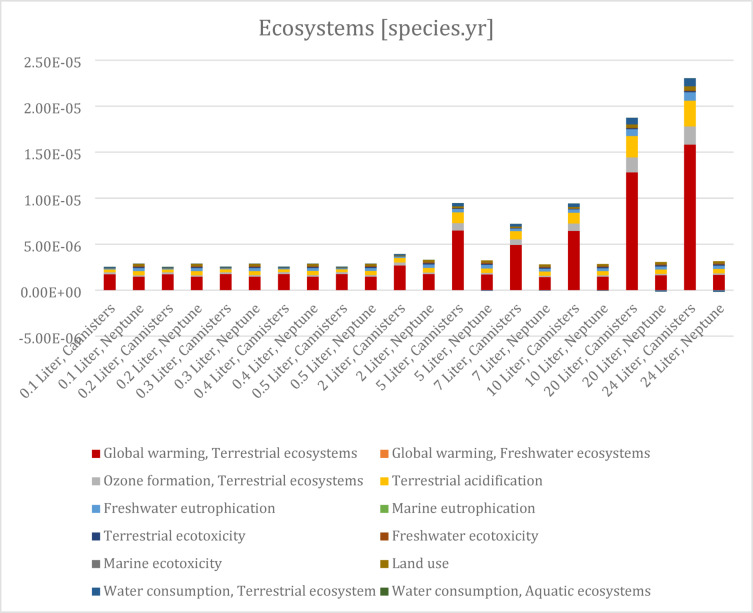
Endpoint results ecosystem.

**Fig. 6:**
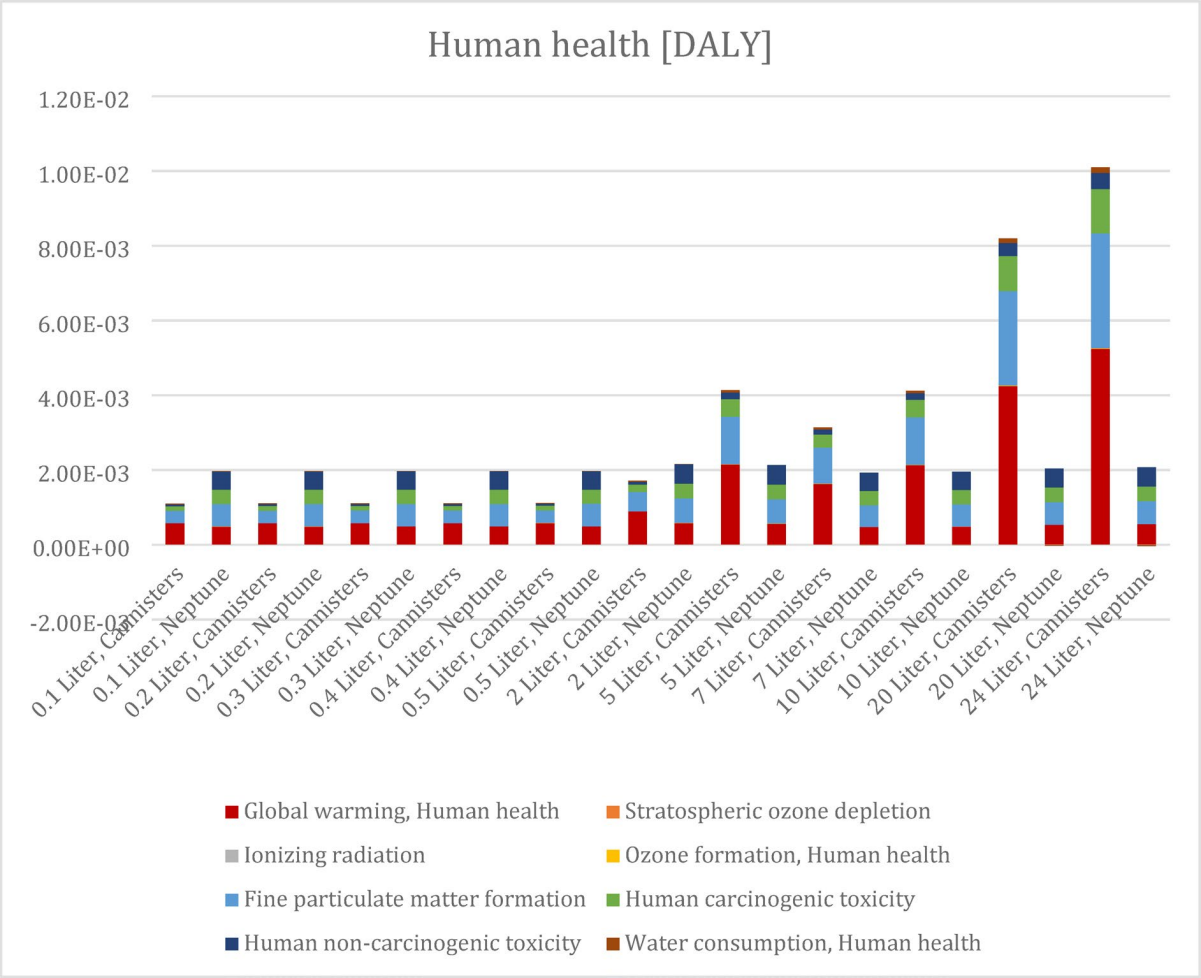
Endpoint results Human Health. DALY: Disability-Adjusted Life Year, represented as a measure used to quantify the overall burden of disease.

**Fig. 7:**
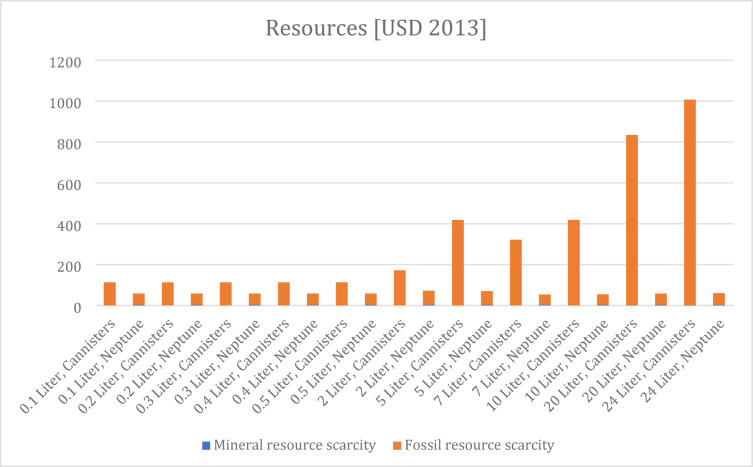
Endpoint results resources.

The 17 midpoint results are converted to three final impact categories, which are ecosystem, human health, and resources. This aggregation helps to compare the relative effects of each impact category. It should be noted that although there is a scientific consensus on the midpoint-categories, there is less consensus on the translation to the endpoint categories (LAP3, 2021). The results are shown in Figs. 5, 6 and 7. Supplementary Table S2 Supplementary Table provides a full overview.

The relative results of the ecosystem endpoint category show that Neptune is a the better performing alternative when fluid volume is two Liters and greater. This is largely attributed to the resulting waste from using canisters. The lower volume categories have a slightly higher impact for Neptune. When looking at the contribution of midpoint results on the ecosystem impact category, it is evident that global warming has the largest effect followed by terrestrial acidification and ozone formation for both alternatives. It should be noted that the Neptune is the better performing alternative for each scenario when global warming and ozone formation are considered (Table 1).

The relative results for human health show a remarkable difference between the alternatives for the larger volume scenarios (5 to 24 L). Furthermore, it may be observed that the canisters alternative performs better for the small volume procedures (0.1 to 0.5 L). Similar to the ecosystem endpoint category, the contribution shows that global warming has the largest effect. This is followed by fine particulate matter formation and human carcinogenic toxicity. In this case, the difference is also largely attributed to the resulting waste from using canisters. Moreover, the use of both single use plastic in the canister liner and the material used to produce the rover is causing an increase in fine particulate matter formation. Finally, the Neptune rover is contributing to the impact in human carcinogenic toxicity, where in the case of canisters the impact originates from the waste treatment process.

The “USD 2013” metric is used to express the environmental impact in monetary terms. By expressing resource scarcity impacts in 2013 U.S. dollars (USD 2013) a standardized economic perspective is shown on environmental impacts. The results for resources show a remarkable difference between the alternatives, though it may be observed that the difference between both alternatives is smaller in the low volume procedures (0.1 to 0.8 L). The main contributor to this endpoint category is fossil resource scarcity.

By aggregating the mid-point results to end-point results it is observed that the Neptune systems is beneficial for resources in each scenario. In case of human health and ecosystems, however, not in all scenarios, the Neptune is beneficial for procedures in particular with larger volumes.

To further illustrate the reduced impact when using the Neptune compared to conventional canisters, the reduction in global warming and fossil resources were translated to number of flights from Amsterdam to Barcelona and barrels of oil, respectively. These calculations have been made based on an amount of 195 kg CO_2_ per flight between Amsterdam and Barcelona and 136 kg per barrel of oil. This resulted in the following values:

**Table:** 

Scenario	Flights	Barrels of oil
0.1 L	0.5	0.7
0.2 L	0.5	0.7
0.3 L	0.5	0.7
0.4 L	0.5	0.7
0.5 L	0.5	0.7
2 L	2.1	1.8
5 L	8.8	6.0
7 L	6.4	4.5
10 L	9.1	6.2
20 L	20.5	13.6
24 L	25.9	16.6

The amount of CO_2_ and fossil resources that can be saved using of Neptune would equal 25 flights from Amsterdam to Barcelona and 16 barrels of oil, respectively. This demonstrates that Neptune has a lower environmental impact compared to canisters.

### Contribution analysis

Contribution analyses as shown in Supplementary Figure S3-S8 Contribution Analysis have been performed to indicate the contribution of each stage of the life cycle of each alternative to the overall environmental profile. This analysis is used to determine hotspots and to compare the importance of different life cycle stages to the result.

From the contribution analysis, it can be noted that in case of the Neptune system the rover is contributing largely to all impact categories. Further analyzing the data shows that this is mainly due to the input of the printed circuit board assemblies and copper-rich material. Furthermore, the cleaning cycle contributes notably to all impact categories, but especially in marine eutrophication and land use. Finally, single use manifolds and electricity requirements related to each use contribute notably to most impact categories.

In the case of the canister alternative it is observed that the treatment of waste plays an important role in all scenarios and impact categories. Furthermore, the use of WIVA containers contributes may depend on the volume of waste streams. Hence, the contribution of the WIVA containers is proportional to the collected volume of fluids. Another important contributor is the use of the single use suction bags. It is assumed that the only reusable suction bag that is used has a volume of 2 L. Hence the suction bag has a bigger contribution in the procedures with small or uneven volumes as the suction bag is not completely filled.

### Sensitivity analysis

One of the uncertainties of this model was the inventory data of the wastewater treatment process. Therefore, a sensitivity analysis is performed over the inventory data related to the wastewater treatment of the collected fluids.

The impact between the baseline and the adapted wastewater treatment process for each category did not result in a notable difference.

A second uncertain factor was the energy mix as this might change in the future. The Netherlands has the ambition to have 100% renewable energy by 2050 (Ministerie van Algemene Zaken, 2022). It was therefore chosen to replace the original Dutch electricity mix with an all-renewable resource energy mix from ecoinvent.

The relative differences shown below for the impact categories were identified to be contributing the most to the endpoint results. A negative value in these figures indicate that the Neptune is the better performing alternative.

**Fig. 8:**
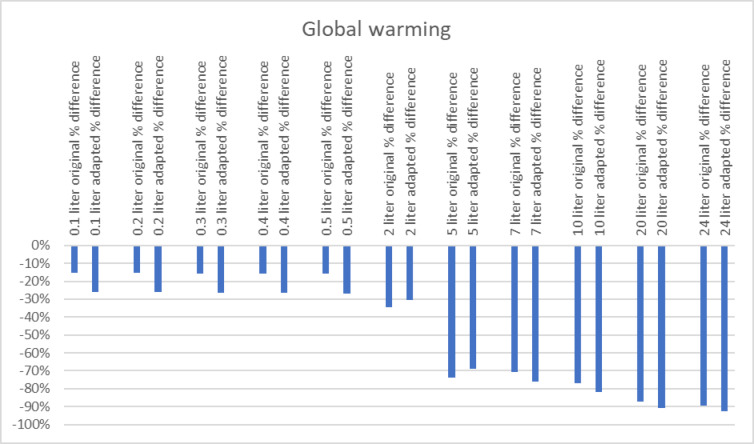
Relative differences global warming between baseline and scenario with adapted waste water process and electricity mix.

When observing global warming in Fig. 8, the difference becomes greater with higher volumes. This means that the Neptune is increasingly positive in terms of global warming compared to the canisters.

### Uncertainty analysis

In line with common practice in comparable LCA studies, a qualitative uncertainty analysis was conducted to evaluate the robustness of the results and identify the parameters most likely to influence the outcome. Several aspects of the life cycle inventory are subject to uncertainty due to limitations in data availability or the use of proxy assumptions. The key sources of uncertainty identified are the Neptune and Rover system composition, electricity use, transportation distances, and waste treatment modelling. Each is discussed below, with illustrative sensitivity results shown in Fig. 8.

Neptune Rover composition:

The main material inputs and component weights for the Neptune system were obtained from manufacturer-provided data. While the material composition was not always available at the subassembly level (e.g., printed circuit boards), key components were included at the material level where possible. Contribution analysis showed that the dominant environmental burdens stem from electricity use, disposable manifolds, and the cleaning cycle. It is therefore unlikely that the exclusion of minor subcomponents significantly affects the overall results.

Electricity use:

Electricity consumption per procedure was modelled using a linear regression based on 34 measurements during procedures. Although this provides an empirical basis, it may not fully capture operational variability (e.g., idle time or non-standard cleaning routines). Nonetheless, the measurements are considered sufficient to represent the main variability such that relative results between Neptune and the canisters remain unaffected. To test the robustness of this assumption, we modelled a scenario with an adapted electricity mix. The outcome, shown in Fig. 8, indicates that variations in electricity parameters do not alter the overall comparative conclusions.

Transport assumptions:

Transport distances and modes were modelled based on information provided by the manufacturer and standard logistics routes from material manufacturing to production. While some variability is expected in actual distribution networks, the contribution of transport to overall impact was relatively small compared to waste treatment and raw material use. Any deviation in transport parameters is therefore unlikely to change the comparative outcomes.

Waste treatment modelling:

The environmental impact of incinerating fluid-filled canisters was modelled using an adapted ecoinvent process based on thermodynamic calculations to account for the energy required to vaporize water. Although based on conservative assumptions, the absence of empirical incineration data may introduce uncertainty. Given the high energy demand of liquid incineration and the exclusion of system inefficiencies, the results for the canister system can be considered conservative, representing a lower-bound estimate. The sensitivity analysis with an adapted wastewater treatment process, visualized in Fig. 8, confirms that even under different modelling assumptions, the relative differences between systems remain robust.

To assess the robustness of the comparative outcomes, we conducted a qualitative uncertainty analysis and tested key parameters (electricity use and wastewater treatment modelling) in sensitivity scenarios. The results of these scenarios are shown in Fig. 8 and are further discussed in the Uncertainty Analysis section.

## Discussion and interpretation

The results from this LCA confirm that the Neptune 3 system is environmentally beneficial compared to traditional canisters for some impact categories. Neptune shows to have a lower environmental impact compared to canisters when global warming, ozone formation (human health), ozone formation (terrestrial ecosystems), fossil resource scarcity and water consumption are considered. In addition, the Neptune system is beneficial for stratospheric ozone depletion, ionizing radiation, fine particulate matter formation, terrestrial acidification and freshwater eutrophication in particular with higher volumes. The Neptune also was proven to be better performing in human carcinogenic toxicity and land use in some of the high-volume scenarios. The Neptune has a larger impact in Marine eutrophication, Terrestrial ecotoxicity, freshwater ecotoxicity, marine ecotoxicity, human non-carcinogenic toxicity, land use and mineral scarcity, with an increasing difference between the alternatives for the small volume categories. By aggregating the mid-point results to end-point results it is observed that the Neptune systems is beneficial for resources in each scenario. In case of human health and ecosystems, the Neptune is beneficial for procedures with larger volumes.

The sensitivity analysis showed that the relative outcomes do not change after the introduction of an adapted wastewater treatment process. The sensitivity analysis confirmed the robustness of the results: even after incorporating an adapted wastewater treatment process, the Neptune system consistently outperformed the canisters in terms of global warming potential across all scenarios, and in fossil resource scarcity under high-volume conditions thereby affirming the reliability of the initial findings.

The second sensitivity analysis, focused on variations in the energy mix, further reinforced Neptune’s environmental advantage. The type of energy source may have different kinds of impact in terms of energy consumption and may change over time. As energy grids evolve toward renewable sources, environmental impacts may shift. The analysis showed a stronger positive response compared to the canisters across a broad range of impact categories, including global warming, stratospheric ozone depletion, ionizing radiation, ozone formation (human health and ecosystems), fine particulate matter formation, freshwater eutrophication, and fossil resource scarcity. While outcomes remained unchanged in scenarios involving less than 7 L, scenarios with higher volumes demonstrated significant additional benefits for the Neptune system in categories such as terrestrial acidification, marine eutrophication, ecotoxicity (terrestrial and marine), human toxicity (carcinogenic and non-carcinogenic), land use, and mineral resource scarcity.

This study evaluated the environmental impact by using the ISO 14,040 and 14,044 life cycle assessment standards. Also, this study considered 17 impact categories (midpoints) defined in the RECIPE model, of which the results were converted into the endpoint categories of human health, ecosystems, and resources using the ReCiPe endpoint characterization model. This is in line with the international standards defined to perform LCAs. Even though LCAs have been conducted in the past on solid waste management, to our knowledge, this is the first LCA study that compares an alternative system for the collection and disposal of surgical fluid with the conventional cannisters. Therefore, comparing the study findings with existing literature on fluid waste management is difficult.

Medical waste decreasing activities are of growing international interest for cost reasons and for reasons to lower the impact on the environment^35–39^. Any new method or product contributing hereto may be regarded as beneficial. While this LCA confirms that Neptune is environmentally beneficial compared to canisters, there are other several advantages that could be relevant for the healthcare sector. The OR staff no longer need to lug collection materials or collecting fluids and divert their resources to patient care and the cooperation/training of colleagues. Fewer collection materials are needed and costs for purchase, storage, transport, and disposal are lower. Not having to lift heavy canisters reduces the risk of back pain and other injuries. Because the Neptune 3 does not require any drainage holes in the OR floor, the work floors are drier, which reduces the risk of slipping and is also more hygienic because no open connection to the sewer in the OR is required. Furthermore, the Neptune 3 system realizes CO_2_ reduction because no transport and combustion are required. Compared to canisters, the Neptune 3 system uses less material resulting in reduced global warming impact and fossil depletion. Nevertheless, the impact of disposing flushing water used during surgical procedures should be further investigated. It could be that the bacteria and viruses disposed in the sewer have an additional, “hidden” impact on the environment that is currently not part of a standard LCA methodology. If so, the standard methodology needs to be expanded.

### Uncertainty analysis

In line with common practice in comparable LCA studies, a qualitative uncertainty analysis was conducted to evaluate the robustness of the results and identify the parameters most likely to influence the outcome. Several aspects of the life cycle inventory are subject to uncertainty due to limitations in data availability or the use of proxy assumptions. The key sources of uncertainty that are identified are the Neptune and Rover, the electricity use, transportation distances and waste treatment modelling were identified as the most uncertain factors in this study. Each uncertainty factor is discussed below.

Neptune rover: The main material inputs and component weights for the Neptune system were obtained from manufacturer-provided data. While the material composition was not always available at the subassembly level (e.g. printed circuit boards), key components were included at the material level where possible. As the contribution analysis indicated that the dominant environmental burdens stem from electricity use, disposable manifolds, and the cleaning cycle, it is unlikely that the exclusion of minor subcomponents significantly affects the overall results.

Electricity use: Electricity consumption per procedure was modeled using a linear regression based on 34 measurements during procedures. Although this provides an empirical basis, it may not fully capture operational variability. This would include for instance idle time and non-standard cleaning routines. Nonetheless, it is assumed that the 34 measurements sufficiently capture the variability such that the relative results between the Neptune and the canisters remain unaffected.

Transport assumptions: Transport distances and modes were modelled based on information given by the manufacturer and standard logistics routes from material manufacturing to production. While some variability is expected in actual distribution networks, the contribution of transport to overall impact was relatively small compared to waste treatment and raw material use. Any deviation in transport parameters is therefore unlikely to change the comparative outcomes.

Waste treatment modelling: The environmental impact of incinerating fluid-filled canisters was modelled using an adapted Ecoinvent process based on thermodynamic calculations to account for the energy required to vaporize water. Although based on conservative assumptions, the absence of empirical incineration data may introduce some uncertainty. Given the high energy demand of liquid incineration and the exclusion of system inefficiencies, the results for the canister system can be considered conservative, representing a lower-bound estimate.

## Limitations

Real-life data used in this LCA was collected from one hospital in The Netherlands (OLVG). In future, data collection from multiple hospitals or a larger sample size could be beneficial to estimate the environmental impact of Neptune 3 in a more robust manner. Also, economic and social factors were not included in this analysis, therefore, life cycle costing (LCC) was not conducted in this study. A LCC would be strongly recommended in further studies to analyse and compare the cost of each phase. Furthermore, it may be helpful for designers when designing new products. The sensitivity analysis was conducted as an (uncertainty) analysis due to uncertainties in the data of the used materials and components. We could not track down all of the materials used in the components. Among other factors, the production of machinery for the manufacturing of face masks and autoclaves are not included in this study since these data were not readily available and therefore outside the system boundary.

## Conclusion

In conclusion, results from this LCA confirm that the Neptune 3 system is environmentally beneficial compared to traditional canisters for some impact categories. Neptune shows to have a lower environmental impact compared to canisters when global warming, ozone formation (human health), ozone formation (terrestrial ecosystems), fossil resource scarcity and water consumption are considered. In addition, the Neptune system is beneficial for, stratospheric ozone depletion, ionizing radiation, fine particulate matter formation, terrestrial acidification and freshwater eutrophication with higher volumes. The Neptune also was proven to be better performing in human carcinogenic toxicity and land use in some of the high-volume scenarios. Overall, the sensitivity scenarios confirm that the comparative results remain robust despite uncertainties in electricity use, transport, and waste treatment modelling, strengthening confidence in the study’s findings. This LCA provides valuable information for policy and decision-makers as the processing of liquids via the Neptune 3 system fits in well with the ambitions of hospitals and governments to treat their surgical waste in an environmentally friendly way. As the Neptune 3 system may be regarded as a medical “liquid suction system” providing collection and disposal of liquids released during surgical procedures through one system, it can play a critical role in tackling the increasing environmental impact of fluid waste management and help healthcare establishment become more eco-responsible.

## Supplementary Information

Below is the link to the electronic supplementary material.

Supplementary Material 1

Supplementary Material 2

Supplementary Material 3

Supplementary Material 4

Supplementary Material 5

## Data Availability

The raw data is available in relation to this manuscript. The authors declare that the data supporting the findings of this study are available within the paper and its Supplementary Information files. Should any raw data files be needed in another format they are available from the corresponding author upon reasonable request. Source data are provided with this paper.
