# Supplementary material for: A life cycle assessment of disposing intra-operative collected fluids, a comparative study between the Neptune 3 versus canister drainage
Source: Sci Rep. 2025 Oct 21;15:36587. doi: 10.1038/s41598-025-20375-1 (PMC12540994; doi:10.1038/s41598-025-20375-1)
Supplement: Supplementary file 2 — Supplementary Material 2 [file 41598_2025_20375_MOESM2_ESM.docx]

# Supplementary Figure S3. Contribution Analysis

The figures show the contribution analysis on different categories

# Supplementary Figure S4. Contribution Analysis Change Canisters

# Supplementary Figure S5. Contribution Analysis Fossil Depletion

# Supplementary Figure S6. Contribution Analysis Fossil Depletion Canisters

# Supplementary Figure S7. Contribution Analysis Water Depletion

# Supplementary Figure S8. Contribution Analysis Water Depletion Canisters
