# Supplementary material for: A life cycle assessment of disposing intra-operative collected fluids, a comparative study between the Neptune 3 versus canister drainage
Source: Sci Rep. 2025 Oct 21;15:36587. doi: 10.1038/s41598-025-20375-1 (PMC12540994; doi:10.1038/s41598-025-20375-1)
Supplement: Supplementary file 3 — Supplementary Material 3 [file 41598_2025_20375_MOESM3_ESM.docx]

# Supplemental figure S1. Protocol.

**Legend: Protocol – LCA**

This study serves to determine the environmental impact of collection surgical liquids during a procedure. To calculate the environmental impact, CO_2_ emissions, and energy- and water usage of the use of the Neptune 3 are considered and compared to the use of conventional cannisters. In this study, the entire product life cycle, which entails material, production, energy and water usage and disposal is considered. The data filled in on this form is a great contribution to this research.


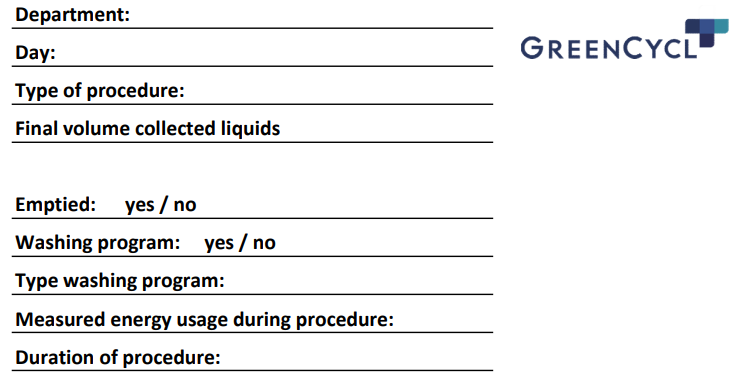


Please email this form to [info@greencycl.nl](mailto:info@greencycl.nl). Thank you for your contribution and input.

Should there be any questions regarding this form or this study, please contact the email above.

**DISCLAIMER**: This form is outside the scope of the General Data Protection Regulation. Please do not enter any personal details
