# Supplementary material for: A life cycle assessment of disposing intra-operative collected fluids, a comparative study between the Neptune 3 versus canister drainage
Source: Sci Rep. 2025 Oct 21;15:36587. doi: 10.1038/s41598-025-20375-1 (PMC12540994; doi:10.1038/s41598-025-20375-1)
Supplement: Supplementary file 4 — Supplementary Material 4 [file 41598_2025_20375_MOESM4_ESM.docx]

# Supplementary Table S1. Functional Units.

**Legend: This table summarizes the included Functional Scenarios**

| Scenario | Number of procedures per year | Functional Unit: intra-operative collected fluids included (L) |
| --- | --- | --- |
| High-volume | 400 | 24 liters |
|  | 400 | 20 liters |
|  | 400 | 10 liters |
|  | 400 | 7 liters |
|  | 800 | 5 liters |
|  | 800 | 2 liters |
| Low-volume | 550 | 0.5 liter |
|  | 550 | 0.4 liter |
|  | 550 | 0.3 liter |
|  | 550 | 0.2 liter |
|  | 550 | 0.1 liter |

A number of scenarios were assessed due to variation in surgical fluids used and collected in different procedural types. The above table defines this range via High- and Low-volume scenarios. The resulting functional unit is defined as providing the collection and disposal of intra-operative fluid waste at these defined fluid volumes and case volumes over seven years of procedures.
