# Supplementary material for: A life cycle assessment of disposing intra-operative collected fluids, a comparative study between the Neptune 3 versus canister drainage
Source: Sci Rep. 2025 Oct 21;15:36587. doi: 10.1038/s41598-025-20375-1 (PMC12540994; doi:10.1038/s41598-025-20375-1)
Supplement: Supplementary file 5 — Supplementary Material 5 [file 41598_2025_20375_MOESM5_ESM.docx]

## Supplementary Figure S1. Inventory Neptune.

Inventory analysis of the Neptune.


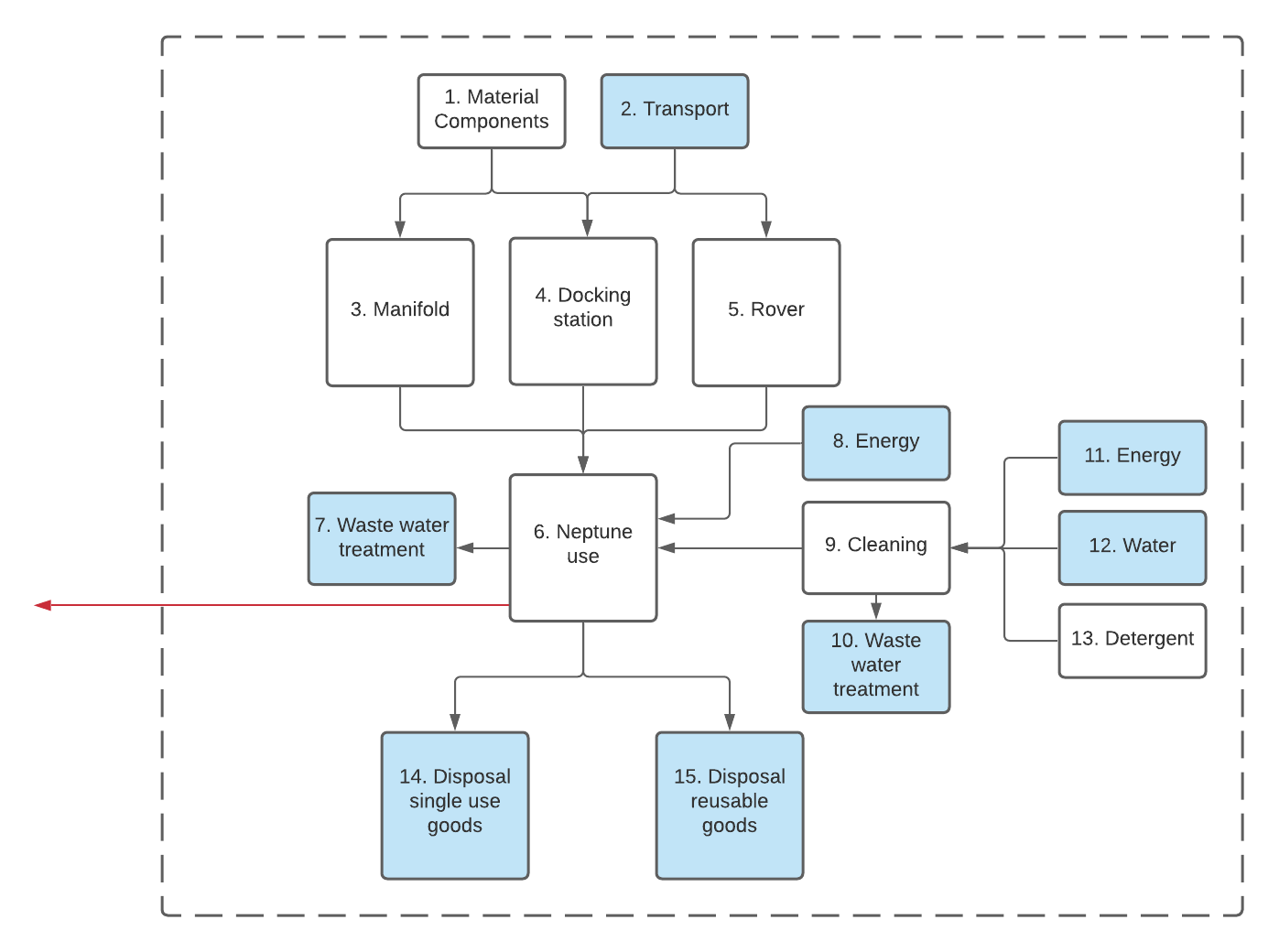


### Material components

The material for the components is obtained from information provided by the supplier in a material database. The database is based on the main material(s) and the weight of each component and can be found in digital appendix DA1 Material Database. The waste caused by each component is included in (3) Manifold, (4) Docking Station and (5) Rover.

### Transport

The transport of the components is partially obtained from information on the production locations of the supplier, the weight of the component and the final assembly location of Stryker, which is Portgage, MI, United States. Here, it was assumed that transport distance below 500 km was performed with a truck. For distances above 500 km, it was assumed that 70% was performed by train and 30% by truck. For unknown production locations, a transport model provided by Ecoinvent (2013) was used. In this transport model it is assumed that, specifically for instruments and appliances for medicine, surgery and dental, the transport consists of 0.293 tkm by truck, 1.112 tkm by sea freight and 0.178 tkm by airfreight. An overview of the transport per component is given in digital appendix DA1 Material Database.

### Supplementary Table S3 Manifold

| **Production of manifold** | | | |
| --- | --- | --- | --- |
| **Economic inputs** | | | |
| **Product** | | **Amount** | **Ecoinvent name** |
| 1 | Polycarbonate | 0.0541 kg | polypropylene, granulate_market for polypropylene, granulate[GLO] |
| 2 | Aircraft transport | 9.63E-6 tkm | transport, freight, aircraft_transport, freight, aircraft, intercontinental[RoW] |
| 3 | Lorry transport | 1.59E-5 tkm | transport, freight, lorry >32 metric ton, EURO4_transport, freight, lorry >32 metric ton, EURO4[RoW] |
| 4 | Sea transport | 6.02E-5 tkm | transport, freight, sea, transoceanic ship_market for transport, freight, sea, transoceanic ship[GLO] |
| **Environmental inputs** | | | |
| **Product** | | **Amount** | **Ecoinvent name** |
|  | N/A |  |  |
| **Economic outputs** | | | |
| **Product** | | **Amount** | **Ecoinvent name** |
|  | 4 port manifold | 1 piece | N/A |
|  | Waste plastic | 0.0541 | waste plastic, mixture_treatment of waste plastic, mixture, municipal incineration [RoW] |
| **Environmental outputs** | | | |
| **Product** | | **Amount** | **Ecoinvent name** |
|  | N/A |  |  |

1. The manifold consists of 0.0521 kg polycarbonate as indicated by the supplier. The component can be found in de material database. The total amount of waste plastic that is produced per manifold is 0.0531 kg.

2 -4. Based on the transport model of Ecoinvent (2013 (0.293 tkm by truck, 1.112 tkm by sea

freight and 0.178 tkm by airfreight per kg) with a weight of 0.0521 kg.

### Supplementary Table S4 Docking Station

| **Production of manifold** | | | |
| --- | --- | --- | --- |
| **Economic inputs** | | | |
| **Product** | | **Amount** | **Ecoinvent name** |
| 1 | Docking station components from material database | 1 piece | N/A |
| **Environmental inputs** | | | |
| **Product** | | **Amount** | **Ecoinvent name** |
|  | N/A |  |  |
| **Economic outputs** | | | |
| **Product** | | **Amount** | **Ecoinvent name** |
|  | Docking station | 1 |  |
|  | Plastic waste | 0.64 kg | waste plastic, mixture_treatment of waste plastic, mixture, municipal incineration[RoW] |
|  | Steel waste | 12.03 kg | scrap steel_treatment of scrap steel, municipal incineration[Europe without Switzerland] |
|  | Miscellaneous waste | 33.03 kg | municipal solid waste_market for municipal solid waste[RoW] |
| **Environmental outputs** | | | |
| **Product** | | **Amount** | **Ecoinvent name** |
|  | N/A |  |  |

1. The docking station consists of 70 components that were obtained from the material database shared by the producer. The total amount of waste is based on the composition of the input material of the components.

### Supplementary Table S5 Rover

| 1. **Production of manifold** | | | |
| --- | --- | --- | --- |
| **Economic inputs** | | | |
| **Product** | | **Amount** | **Ecoinvent name** |
| 1 | Docking station components from material database | 1 piece | N/A |
| **Environmental inputs** | | | |
| **Product** | | **Amount** | **Ecoinvent name** |
|  | N/A |  |  |
| **Economic outputs** | | | |
| **Product** | | **Amount** | **Ecoinvent name** |
| 2 | Plastic waste | 73.83 kg | waste plastic, mixture_treatment of waste plastic, mixture, municipal incineration[RoW] |
| 3 | Steel waste | 30.00 kg | scrap steel_treatment of scrap steel, municipal incineration[Europe without Switzerland] |
| 4 | Miscellaneous waste | 9.59 kg | municipal solid waste_market for municipal solid waste[RoW] |
| **Environmental outputs** | | | |
| **Product** | | **Amount** | **Ecoinvent name** |
|  | N/A |  |  |

1. The rover consists of 195 components that can be found in the material database. The total amount of waste is based on the composition of the input material of the components.

### Supplementary Table S6 Neptune use

In this study, a total of nine usage scenarios are included, which are:

|  | Number of procedures | Average liters |
| --- | --- | --- |
| 24 liter scenario | 400 | 24 liters |
| 20 liter scenario | 400 | 20 liters |
| 10 liter scenario | 400 | 10 liters |
| 7 liter scenario | 400 | 7 liters |
| 5 liter scenario | 800 | 5 liters |
| 2 liter scenario | 800 | 2 liters |
| 500CC scen | 550 | 0.5 liter |
| 400CC scen | 550 | 0.4 liter |
| 300CC scen | 550 | 0.3 liter |
| 200CC scen | 550 | 0.2 liter |
| 100CC scen | 550 | 0.1 liter |

### Supplementary Table S7 25 liter scenario

| **Neptune use – 25 liter** | | | |
| --- | --- | --- | --- |
| **Economic inputs** | | | |
| **Product** | | **Amount** | **Ecoinvent name** |
| 1 | Cleaning procedure | 250 | N/A |
| 2 | Energy use | 9.24 kWh | electricity, low voltage_market for electricity, low voltage[NL] |
| 3 | Manifold | 400 units | N/A |
| 4 | Docking station | 0.036 units | N/A |
| 5 | Rover | 0.14 | N/A |
| **Environmental inputs** | | | |
| **Product** | | **Amount** | **Ecoinvent name** |
|  | N/A |  |  |
| **Economic outputs** | | | |
| **Product** | | **Amount** | **Ecoinvent name** |
| 6 | Waste water treatment | 10 m^3^ | wastewater, average_treatment of wastewater, average, capacity 1E9l/year[Europe without Switzerland] |
| 7 | 1 year of use |  | N/A |
| **Environmental outputs** | | | |
| **Product** | | **Amount** | **Ecoinvent name** |
|  | N/A |  |  |

1. Based on information provided by Stryker, it is assumed that for each scenario 250 cleaning cycles are conducted per year.
2. An energy use of 0.07 kWh was measured over an hour. This means that, assuming that the Neptune is 20 mins effectively used during a procedure and 400 procedures per year, a total energy consumption of 9.24 kWh is considered.
3. 1 per procedure. Hence, 400 manifolds are required
4. 0.036 (1 per 4 Neptunes, lifespan 7 year)
5. 1 on the department with a lifespan of 7 year. Hence 0.14
6. liquid from procedures (400*24) = 96000 liter per year, 9.6 m^3^ per year.

### Supplementary Table S8 20 liter scenario

| **Neptune use – 20 liter** | | | |
| --- | --- | --- | --- |
| **Economic inputs** | | | |
| **Product** | | **Amount** | **Ecoinvent name** |
| 1 | Cleaning procedure | 250 | N/A |
| 2 | Energy use | 9.24 kWh | electricity, low voltage_market for electricity, low voltage[NL] |
| 3 | Manifold | 400 units | N/A |
| 4 | Docking station | 0.036 units | N/A |
| 5 | Rover | 0.14 | N/A |
| **Environmental inputs** | | | |
| **Product** | | **Amount** | **Ecoinvent name** |
|  | N/A |  |  |
| **Economic outputs** | | | |
| **Product** | | **Amount** | **Ecoinvent name** |
| 6 | Waste water treatment | 8 m^3^ | wastewater, average_treatment of wastewater, average, capacity 1E9l/year[Europe without Switzerland] |
| 7 | 1 year of use |  | N/A |
| **Environmental outputs** | | | |
| **Product** | | **Amount** | **Ecoinvent name** |
|  | N/A |  |  |

1. Based on information provided by Stryker, it is assumed that for each scenario 250 cleaning cycles are conducted per year.
2. An energy use of 0.07 kWh was measured over an hour. This means that, assuming that the Neptune is 20 mins effectively used during a procedure and 400 procedures per year, a total energy consumption of 9.24 kWh is considered.
3. 1 per procedure. Hence, 400 manifolds are required
4. 0.036 (1 per 4 Neptunes, lifespan 7 year)
5. 1 on the department with a lifespan of 7 year. Hence 0.14
6. liquid from procedures (400*20) = 4000 liter per year, 8 m^3^ per year.

### Supplementary Table S9 10 liter scenario

| **Neptune use – 10 liter** | | | |
| --- | --- | --- | --- |
| **Economic inputs** | | | |
| **Product** | | **Amount** | **Ecoinvent name** |
| 1 | Cleaning procedure | 250 | N/A |
| 2 | Energy use | 9.24 kWh | electricity, low voltage_market for electricity, low voltage[NL] |
| 3 | Manifold | 400 units | N/A |
| 4 | Docking station | 0.036 units | N/A |
| 5 | Rover | 0.14 | N/A |
| **Environmental inputs** | | | |
| **Product** | | **Amount** | **Ecoinvent name** |
|  | N/A |  |  |
| **Economic outputs** | | | |
| **Product** | | **Amount** | **Ecoinvent name** |
| 6 | Waste water treatment | 4 m^3^ | wastewater, average_treatment of wastewater, average, capacity 1E9l/year[Europe without Switzerland] |
| 7 | 1 year of use |  | N/A |
| **Environmental outputs** | | | |
| **Product** | | **Amount** | **Ecoinvent name** |
|  | N/A |  |  |

1. Based on information provided by Stryker, it is assumed that for each scenario 250 cleaning cycles are conducted per year.
2. An energy use of 0.07 kWh was measured over an hour. This means that, assuming that the Neptune is 20 mins effectively used during a procedure and 400 procedures per year, a total energy consumption of 9.24 kWh is considered.
3. 1 per procedure. Hence, 400 manifolds are required
4. 0.036 (1 per 4 Neptunes, lifespan 7 year)
5. 1 on the department with a lifespan of 7 year. Hence 0.14
6. liquid from procedures (400*10) = 4000 liter per year, 4 m^3^ per year.

### Supplementary Table S10 liter scenario

| **Neptune use – 7 liter** | | | |
| --- | --- | --- | --- |
| **Economic inputs** | | | |
| **Product** | | **Amount** | **Ecoinvent name** |
| 1 | Cleaning procedure | 250 | N/A |
| 2 | Energy use | 18.48  kWh | electricity, low voltage_market for electricity, low voltage[NL] |
| 3 | Manifold | 800 units | N/A |
| 4 | Docking station | 0.036 units | N/A |
| 5 | Rover | 0.14 | N/A |
| **Environmental inputs** | | | |
| **Product** | | **Amount** | **Ecoinvent name** |
|  | N/A |  |  |
| **Economic outputs** | | | |
| **Product** | | **Amount** | **Ecoinvent name** |
| 6 | Waste water treatment | 4 m^3^ | wastewater, average_treatment of wastewater, average, capacity 1E9l/year[Europe without Switzerland] |
| 7 | 1 year of use |  | N/A |
| **Environmental outputs** | | | |
| **Product** | | **Amount** | **Ecoinvent name** |
|  | N/A |  |  |

1. Based on information provided by Stryker, it is assumed that for each scenario 250 cleaning cycles are conducted per year.
2. An energy use of 0.07 kWh was measured over an hour. This means that, assuming that the Neptune is 20 mins effectively used during a procedure and 800 procedures per year, a total energy consumption of 18.48 kWh is considered.
3. 1 per procedure. Hence, 800 manifolds are required
4. 0.072 (1 per 4 Neptunes, lifespan 7 year))
5. 1 on the department with a lifespan of 7 year. Hence 0.14
6. liquid from procedures (800*5) = 4000 liter per year, 4 m^3^ per year.

### Supplementary Table S11 5 liter scenario

| **Neptune use – 5 liter** | | | |
| --- | --- | --- | --- |
| **Economic inputs** | | | |
| **Product** | | **Amount** | **Ecoinvent name** |
| 1 | Cleaning procedure | 250 | N/A |
| 2 | Energy use | 607.2 kWh | electricity, low voltage_market for electricity, low voltage[NL] |
| 3 | Manifold | 800 units | N/A |
| 4 | Docking station | 0.036 units | N/A |
| 5 | Rover | 0.14 | N/A |
| **Environmental inputs** | | | |
| **Product** | | **Amount** | **Ecoinvent name** |
|  | N/A |  |  |
| **Economic outputs** | | | |
| **Product** | | **Amount** | **Ecoinvent name** |
| 6 | Waste water treatment | 1.6 m^3^ | wastewater, average_treatment of wastewater, average, capacity 1E9l/year[Europe without Switzerland] |
| 7 | 1 year of use |  | N/A |
| **Environmental outputs** | | | |
| **Product** | | **Amount** | **Ecoinvent name** |
|  | N/A |  |  |

1. Based on information provided by Stryker, it is assumed that for each scenario 250 cleaning cycles are conducted per year.
2. An energy use of 0.07 kWh was measured over an hour. This means that, assuming that the Neptune is 20 mins effectively used during a procedure and 800 procedures per year, a total energy consumption of 18.48 kWh is considered.
3. 1 per procedure. Hence, 800 manifolds are required
4. 0.036 (1 per 4 Neptunes, lifespan 7 year))
5. 1 on the department with a lifespan of 7 year. Hence 0.14
6. liquid from procedures (800*2) = 1600 liter per year, 1.6 m^3^ per year.

### Supplementary Table S12 2 liter scenario

| **Neptune use – 2 liter** | | | |
| --- | --- | --- | --- |
| **Economic inputs** | | | |
| **Product** | | **Amount** | **Ecoinvent name** |
| 1 | Cleaning procedure | 250 | N/A |
| 2 | Energy use | 9.24 kWh | electricity, low voltage_market for electricity, low voltage[NL] |
| 3 | Manifold | 400 units | N/A |
| 4 | Docking station | 0.036 units | N/A |
| 5 | Rover | 0.14 | N/A |
| **Environmental inputs** | | | |
| **Product** | | **Amount** | **Ecoinvent name** |
|  | N/A |  |  |
| **Economic outputs** | | | |
| **Product** | | **Amount** | **Ecoinvent name** |
| 6 | Waste water treatment | 2.8 m^3^ | wastewater, average_treatment of wastewater, average, capacity 1E9l/year[Europe without Switzerland] |
| 7 | 1 year of use |  | N/A |
| **Environmental outputs** | | | |
| **Product** | | **Amount** | **Ecoinvent name** |
|  | N/A |  |  |

1. Based on information provided by Stryker, it is assumed that for each scenario 250 cleaning cycles are conducted per year.
2. An energy use of 0.07 kWh was measured over an hour. This means that, assuming that the Neptune is 20 mins effectively used during a procedure and 400 procedures per year, a total energy consumption of 9.24 kWh is considered.
3. 1 per procedure. Hence, 400 manifolds are required
4. 0.036 (1 per 4 Neptunes, lifespan 7 year))
5. 1 on the department with a lifespan of 7 year. Hence 0.14
6. liquid from procedures (400*7) = 2800 liter per year, 2.8 m^3^ per year.

### Supplementary Table S13 500CC scenario

| **Neptune use – 500CC** | | | |
| --- | --- | --- | --- |
| **Economic inputs** | | | |
| **Product** | | **Amount** | **Ecoinvent name** |
| 1 | Cleaning procedure | 250 | N/A |
| 2 | Energy use | 12.71 kWh | electricity, low voltage_market for electricity, low voltage[NL] |
| 3 | Manifold | 550 units | N/A |
| 4 | Docking station | 0.036 units | N/A |
| 5 | Rover | 0.14 | N/A |
| **Environmental inputs** | | | |
| **Product** | | **Amount** | **Ecoinvent name** |
|  | N/A |  |  |
| **Economic outputs** | | | |
| **Product** | | **Amount** | **Ecoinvent name** |
| 6 | Waste water treatment | 2.25 m^3^ | wastewater, average_treatment of wastewater, average, capacity 1E9l/year[Europe without Switzerland] |
| 7 | 1 year of use |  | N/A |
| **Environmental outputs** | | | |
| **Product** | | **Amount** | **Ecoinvent name** |
|  | N/A |  |  |

1. Based on information provided by Stryker, it is assumed that for each scenario 250 cleaning cycles are conducted per year.
2. An energy use of 0.07 kWh was measured over an hour. This means that, assuming that the Neptune is 20 mins effectively used during a procedure and 550 procedures per year, a total energy consumption of 12.71 kWh is considered.
3. 1 per procedure. Hence, 550 manifolds are required
4. 0.036 (1 per 4 Neptunes, lifespan 7 year))
5. 1 on the department with a lifespan of 7 year. Hence 0.14
6. liquid from procedures (550*0.5) = 225 liter per year, 0.225 m^3^ per year.

### Supplementary Table S14 400cc scenario

| **Neptune use – 400CC** | | | |
| --- | --- | --- | --- |
| **Economic inputs** | | | |
| **Product** | | **Amount** | **Ecoinvent name** |
| 1 | Cleaning procedure | 250 | N/A |
| 2 | Energy use | 12.71 kWh | electricity, low voltage_market for electricity, low voltage[NL] |
| 3 | Manifold | 550 units | N/A |
| 4 | Docking station | 0.036 units | N/A |
| 5 | Rover | 0.14 | N/A |
| **Environmental inputs** | | | |
| **Product** | | **Amount** | **Ecoinvent name** |
|  | N/A |  |  |
| **Economic outputs** | | | |
| **Product** | | **Amount** | **Ecoinvent name** |
| 6 | Waste water treatment | 2.2 m^3^ | wastewater, average_treatment of wastewater, average, capacity 1E9l/year[Europe without Switzerland] |
| 7 | 1 year of use |  | N/A |
| **Environmental outputs** | | | |
| **Product** | | **Amount** | **Ecoinvent name** |
|  | N/A |  |  |

1. Based on information provided by Stryker, it is assumed that for each scenario 250 cleaning cycles are conducted per year.
2. An energy use of 0.07 kWh was measured over an hour. This means that, assuming that the Neptune is 20 mins effectively used during a procedure and 550 procedures per year, a total energy consumption of 12.71 kWh is considered.
3. 1 per procedure. Hence, 550 manifolds are required
4. 0.036 (1 per 4 Neptunes, lifespan 7 year))
5. 1 on the department with a lifespan of 7 year. Hence 0.14
6. liquid from procedures (550*0.4) = 220 liter per year, 0.22 m^3^ per year.

### Supplementary Table S15 300CC scenario

| **Neptune use – 300CC** | | | |
| --- | --- | --- | --- |
| **Economic inputs** | | | |
| **Product** | | **Amount** | **Ecoinvent name** |
| 1 | Cleaning procedure | 250 | N/A |
| 2 | Energy use | 12.71 kWh | electricity, low voltage_market for electricity, low voltage[NL] |
| 3 | Manifold | 550 units | N/A |
| 4 | Docking station | 0.036 units | N/A |
| 5 | Rover | 0.14 | N/A |
| **Environmental inputs** | | | |
| **Product** | | **Amount** | **Ecoinvent name** |
|  | N/A |  |  |
| **Economic outputs** | | | |
| **Product** | | **Amount** | **Ecoinvent name** |
| 6 | Waste water treatment | 1.65 m^3^ | wastewater, average_treatment of wastewater, average, capacity 1E9l/year[Europe without Switzerland] |
| 7 | 1 year of use |  | N/A |
| **Environmental outputs** | | | |
| **Product** | | **Amount** | **Ecoinvent name** |
|  | N/A |  |  |

1. Based on information provided by Stryker, it is assumed that for each scenario 250 cleaning cycles are conducted per year.
2. An energy use of 0.07 kWh was measured over an hour. This means that, assuming that the Neptune is 20 mins effectively used during a procedure and 550 procedures per year, a total energy consumption of 12.71 kWh is considered.
3. 1 per procedure. Hence, 550 manifolds are required
4. 0.036 (1 per 4 Neptunes, lifespan 7 year))
5. 1 on the department with a lifespan of 7 year. Hence 0.14
6. liquid from procedures (550*0.3) = 165 liter per year, 0.165 m^3^ per year.

### Supplementary Table S16 200CC scenario

| **Neptune use – 200CC** | | | |
| --- | --- | --- | --- |
| **Economic inputs** | | | |
| **Product** | | **Amount** | **Ecoinvent name** |
| 1 | Cleaning procedure | 250 | N/A |
| 2 | Energy use | 12.71 kWh | electricity, low voltage_market for electricity, low voltage[NL] |
| 3 | Manifold | 550 units | N/A |
| 4 | Docking station | 0.036 units | N/A |
| 5 | Rover | 0.14 | N/A |
| **Environmental inputs** | | | |
| **Product** | | **Amount** | **Ecoinvent name** |
|  | N/A |  |  |
| **Economic outputs** | | | |
| **Product** | | **Amount** | **Ecoinvent name** |
| 6 | Waste water treatment | 1.1 m^3^ | wastewater, average_treatment of wastewater, average, capacity 1E9l/year[Europe without Switzerland] |
| 7 | 1 year of use |  | N/A |
| **Environmental outputs** | | | |
| **Product** | | **Amount** | **Ecoinvent name** |
|  | N/A |  |  |

1. Based on information provided by Stryker, it is assumed that for each scenario 250 cleaning cycles are conducted per year.
2. An energy use of 0.07 kWh was measured over an hour. This means that, assuming that the Neptune is 20 mins effectively used during a procedure and 550 procedures per year, a total energy consumption of 12.71 kWh is considered.
3. 1 per procedure. Hence, 550 manifolds are required
4. 0.036 (1 per 4 Neptunes, lifespan 7 year))
5. 1 on the department with a lifespan of 7 year. Hence 0.14
6. liquid from procedures (550*0.2) = 110 liter per year, 0.11 m^3^ per year.

### Supplementary Table S17 100CC scenario

| **Neptune use – 200CC** | | | |
| --- | --- | --- | --- |
| **Economic inputs** | | | |
| **Product** | | **Amount** | **Ecoinvent name** |
| 1 | Cleaning procedure | 250 | N/A |
| 2 | Energy use | 12.71 kWh | electricity, low voltage_market for electricity, low voltage[NL] |
| 3 | Manifold | 550 units | N/A |
| 4 | Docking station | 0.036 units | N/A |
| 5 | Rover | 0.14 | N/A |
| **Environmental inputs** | | | |
| **Product** | | **Amount** | **Ecoinvent name** |
|  | N/A |  |  |
| **Economic outputs** | | | |
| **Product** | | **Amount** | **Ecoinvent name** |
| 6 | Waste water treatment | 0.55 m^3^ | wastewater, average_treatment of wastewater, average, capacity 1E9l/year[Europe without Switzerland] |
| 7 | 1 year of use |  | N/A |
| **Environmental outputs** | | | |
| **Product** | | **Amount** | **Ecoinvent name** |
|  | N/A |  |  |

1. Based on information provided by Stryker, it is assumed that for each scenario 250 cleaning cycles are conducted per year.
2. An energy use of 0.07 kWh was measured over an hour. This means that, assuming that the Neptune is 20 mins effectively used during a procedure and 550 procedures per year, a total energy consumption of 12.71 kWh is considered.
3. 1 per procedure. Hence, 550 manifolds are required
4. 0.036 (1 per 4 Neptunes, lifespan 7 year))
5. 1 on the department with a lifespan of 7 year. Hence 0.14
6. liquid from procedures (550*0.1) = 55 liter per year, 0.055 m^3^ per year.

### Waste water treatment

The inventory data used to model the treatment of the intra-operative collected fluids is obtained from Ecoinvent.

### Energy

The inventory data used to model the energy that is used to operate the Neptune and docking station is obtained from Ecoinvent.

### Supplementary Table S18 Cleaning cycle (20 mins)

| **Cleaning cycle** | | | |
| --- | --- | --- | --- |
| **Economic inputs** | | | |
| **Product** | | **Amount** | **Ecoinvent name** |
| 1 | Water | 34 L | tap water_market for tap water[Europe without Switzerland] |
| 2 | Energy use | 0.92 kWh | electricity, low voltage_market for electricity, low voltage[NL] |
| 3 | Detergent | 0.09L | N/A |
| **Environmental inputs** | | | |
| **Product** | | **Amount** | **Ecoinvent name** |
|  | N/A |  |  |
| **Economic outputs** | | | |
| **Product** | | **Amount** | **Ecoinvent name** |
| 4 | Waste water treatment | 0.034 m^3^ | wastewater, average_treatment of wastewater, average, capacity 1E9l/year[Europe without Switzerland] |
| 5 | Cleaning Cycle with the Neptune |  | N/A |
| **Environmental outputs** | | | |
| **Product** | | **Amount** | **Ecoinvent name** |
|  | N/A |  |  |

1. 34L as mentioned in the Instructions for Use of the Neptune
2. Energy = m x c x ∆T = 34 x 4186 J/kg x 23.3 = 3316149.2 J = 0.92 kWh
3. Based on information provided by Stryker
4. Used input water is equal to wastewater

## Waste water treatment

The inventory data used to model the treatment of waste water from a cleaning cycle is obtained from Ecoinvent.

## Energy

The inventory data used to model the energy that is used to warm the water for a cleaning cycle is obtained from ecoinvent.

## Water

The inventory data used to model the water that is used in a cleaning cycle is obtained from Ecoinvent.

## Detergent

### Supplementary Table S19 Cleaning cycle

| **Cleaning cycle** | | | |
| --- | --- | --- | --- |
| **Economic inputs** | | | |
| **Product** | | **Amount** | **Ecoinvent name** |
| 1 | Water | 0.7 L | tap water_market for tap water[Europe without Switzerland] |
| 2 | Alcohol Ethoxylate(s) | 0.05 kg | ethoxylated alcohol (AE11)_market for ethoxylated alcohol (AE11)[GLO] |
| 3 | Sodium Lauryl Sulphate | 0.15 kg | alkyl sulphate (C12-14)_market for alkyl sulphate (C12-14)[GLO] |
| 4 | Alkyloxypolyethyleneoxyethanol | 0.1 kg | ethoxylated alcohol (AE7)_market for ethoxylated alcohol (AE7)[GLO] |
| **Environmental inputs** | | | |
| **Product** | | **Amount** | **Ecoinvent name** |
|  | N/A |  |  |
| **Economic outputs** | | | |
| **Product** | | **Amount** | **Ecoinvent name** |
| 5 | Detergent | 1 kg |  |
| **Environmental outputs** | | | |
| **Product** | | **Amount** | **Ecoinvent name** |
|  | N/A |  |  |

1. Based on information provided by supplier
2. Based on information provided by supplier
3. Based on information provided by supplier
4. Based on information provided by supplier

Supplementary Figure S2 Inventory Cannisters


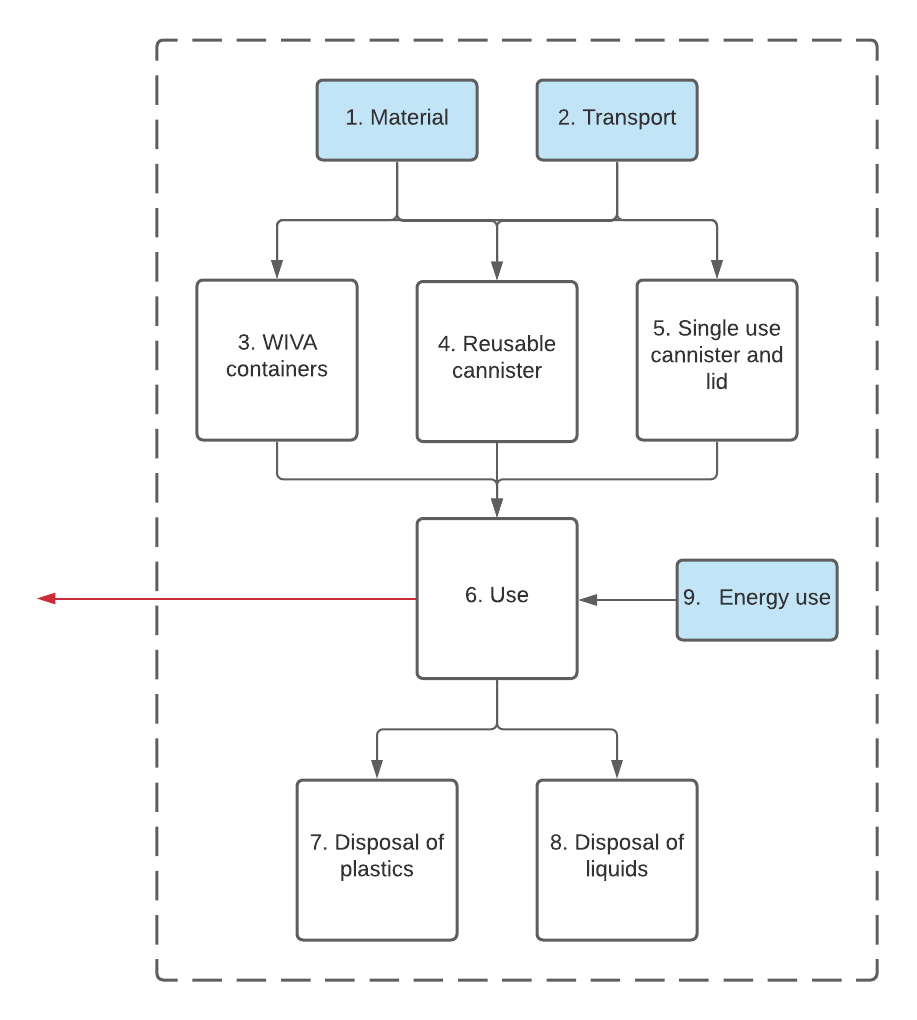


## Material

The inventory data for the material that is used to produce the WIVA containers, Reusable cannister and single us cannister and lid is directly obtained from the Ecoinvent database.

## Transport

The inventory data for the transport modes that is used to produce the WIVA containers, Reusable cannister and single us cannister and lid is directly obtained from the Ecoinvent database.

### Supplementary Table S20 WIVA containers

| **WIVA container** | | | |
| --- | --- | --- | --- |
| **Economic inputs** | | | |
| **Product** | | **Amount** | **Ecoinvent name** |
| 1 | Polyproylene | 1.08 kg | polypropylene, granulate_market for polypropylene, granulate[GLO] |
| 2 | Sea freight | 23.04 tkm | transport, freight, sea, transoceanic ship with reefer, freezing_market for transport, freight, sea, transoceanic ship with reefer, freezing[GLO] |
| 3 | Road freight | 0.78 tkm | transport, freight, lorry 16-32 metric ton, EURO6_transport, freight, lorry 16-32 metric ton, EURO6[RoW] |
| **Environmental inputs** | | | |
| **Product** | | **Amount** | **Ecoinvent name** |
|  | N/A |  |  |
| **Economic outputs** | | | |
| **Product** | | **Amount** | **Ecoinvent name** |
| 4 | Plastic waste | 1.08 | waste plastic, mixture_treatment of waste plastic, mixture, municipal incineration[RoW] |
| 5 | WIVA container (30L) | 1 unit |  |
| **Environmental outputs** | | | |
| **Product** | | **Amount** | **Ecoinvent name** |
|  | N/A |  |  |

1. Based on information of the supplier, it was established that a WIVA container has a weight of 1.08 kg and consists mainly of polypropylene.

2+3: It has been assumed that the containers are produced in Shanghai, China and that the

following transport route was applicable to the hospital in the Netherlands:

- Distance port of Shanghai to Southampton, UK = 21694.33 km
- Distance manufacturer to port of Shanghai = 15.9 km
- Port of Southampton to UK distributor = 92.22 km
- UK distributor to Netherlands = 611.55 km

1. The amount of input material of the WIVA container is incinerated after a single use.

### Supplementary Table S21 Reusable Cannister

| **Reusable Cannister** | | | |
| --- | --- | --- | --- |
| **Economic inputs** | | | |
| **Product** | | **Amount** | **Ecoinvent name** |
| 1 | Polycarbonate | 0.36 kg | polycarbonate_market for polycarbonate[GLO] |
| 2 | Sea freight | 7.81 tkm | transport, freight, sea, transoceanic ship with reefer, freezing_market for transport, freight, sea, transoceanic ship with reefer, freezing[GLO] |
| 3 | Road freight | 0.26 tkm | transport, freight, lorry 16-32 metric ton, EURO6_transport, freight, lorry 16-32 metric ton, EURO6[RoW] |
| **Environmental inputs** | | | |
| **Product** | | **Amount** | **Ecoinvent name** |
|  | N/A |  |  |
| **Economic outputs** | | | |
| **Product** | | **Amount** | **Ecoinvent name** |
| 4 | Plastic waste | 1.08 | waste plastic, mixture_treatment of waste plastic, mixture, municipal incineration[RoW] |
| 5 | Reusable cannister | 1 unit |  |
| **Environmental outputs** | | | |
| **Product** | | **Amount** | **Ecoinvent name** |
|  | N/A |  |  |

1. Based on information of the supplier (Bemis, Wisconsin, USA) it was established that a reusable canister has a weight of 0.36 kg and consists mainly of polycarbonate.

2+3: It has been assumed that the canisters are produced in Shanghai, China and that the

following transport route was applicable to the hospital in the Netherlands:

- Distance port of Shanghai to Southampton, UK = 21694.33 km
- Distance manufacturer to port of Shanghai = 15.9 km
- Port of Southampton to UK distributor = 92.22 km
- UK distributor to Netherlands = 611.55 km

1. The amount of input material of the canister is incinerated after the lifespan.

### Supplementary Table S22 Single use Cannister & Lid

| **WIVA container** | | | |
| --- | --- | --- | --- |
| **Economic inputs** | | | |
| **Product** | | **Amount** | **Ecoinvent name** |
| 1 |  |  |  |
| 2 | Sea freight | 3.69 tkm | transport, freight, sea, transoceanic ship with reefer, freezing_market for transport, freight, sea, transoceanic ship with reefer, freezing[GLO] |
| 3 | Road freight | 0.12 tkm | transport, freight, lorry 16-32 metric ton, EURO6_transport, freight, lorry 16-32 metric ton, EURO6[RoW] |
| 4 | Polyethylene cannister | 0.17 kg | polyethylene, high density, granulate_polyethylene, high density, granulate, recycled to generic market for high density PE granulate[RoW] |
| **Environmental inputs** | | | |
| **Product** | | **Amount** | **Ecoinvent name** |
|  | N/A |  |  |
| **Economic outputs** | | | |
| **Product** | | **Amount** | **Ecoinvent name** |
| 5 | Single use cannister | 1 unit |  |
| 6 | Waste | 0.059 kg | waste plastic, mixture_treatment of waste plastic, mixture, municipal incineration[RoW] |
| **Environmental outputs** | | | |
| **Product** | | **Amount** | **Ecoinvent name** |
|  | N/A |  |  |

1. By weighting a single use suction bag and lid of Serres (Massachusetts, USA), it was established that a lid of a single use cannister has a weight of 0.014 kg and consists mainly of polypropylene.

2+3: It has been assumed that the containers are produced in Shanghai, China and that the

following transport route was applicable to the hospital in the Netherlands:

- Distance port of Shanghai to Southampton, UK = 21694.33 km
- Distance manufacturer to port of Shanghai = 15.9 km
- Port of Southampton to UK distributor = 92.22 km
- UK distributor to Netherlands = 611.55 km

1. By weighting a single use suction bag and lid of Serres (Massachusetts, USA), it was established that a single use cannister has a weight of 0.17 kg and consists mainly of polypropylene.

### Supplementary Table S23 Use

In this study, a total of nine usage scenarios are included, which are:

|  | Number of procedures | Average liters |
| --- | --- | --- |
| Urology | 400 | 10 liter |
| Orthopedics | 800 | 5 liter |
| Gynecology | 400 | 7 liter |
| Low scenario | 800 | 2 liter |
| 500CC scen | 550 | 0.5 liter |
| 400CC scen | 550 | 0.4 liter |
| 300CC scen | 550 | 0.3 liter |
| 200CC scen | 550 | 0.2 liter |
| 100CC scen | 550 | - 1. liter |

### Supplementary Table S24 25 Liter scenario

| **25 liter scenario** | | | |
| --- | --- | --- | --- |
| **Economic inputs** | | | |
| **Product** | | **Amount** | **Ecoinvent name** |
| 1 | Reusable cannister | 12 units | N/A |
| 2 | Single use cannister | 4800 units | N/A |
| 3 | WIVA container | 320 | N/A |
| 4 | Electricity use | 43.56 kWh | electricity, low voltage_market for electricity, low voltage[NL] |
| **Environmental inputs** | | | |
| **Product** | | **Amount** | **Ecoinvent name** |
|  | N/A |  |  |
| **Economic outputs** | | | |
| **Product** | | **Amount** | **Ecoinvent name** |
| 5 | Incinerated intra-operative collected fluids | 9600 liter | N/A |
| 6 | 1 year of use |  |  |
| 7 | Incineration of plastic | 983.64 kg | waste plastic, mixture_treatment of waste plastic, mixture, municipal incineration[RoW] |
| **Environmental outputs** | | | |
| **Product** | | **Amount** | **Ecoinvent name** |
|  | N/A |  |  |

1. 12 per procedure as the reusable cannisters have a volume of 2 liter.
2. 12 per procedure with a volume of 2 liter. Hence, 4800 single use cannisters
3. Assumed that 0.80 container is required per procedure by dividing the volume with the liquids (24/30). Thus, a total of 320 containers.
4. Based on data sheet of fluid management system of Serres (n.d.) that uses a separate machine for fluid management with the use of cannisters (6x6L). This machine uses 330 VA, which equals 0.33 kW. With a duration 2 hours of which the machine is effectively used for 20 minutes, the total energy consumption is: 0.33*0.33*400 = 43.56 kWh.
5. The intra-operative collected fluids are directly incinerated together with the cannisters and WIVA container. In a year, 400 procedures are performed with an average of 24 liters. Thus, 9600 liter is incinerated.
6. n/a
7. Incineration of single-use cannister: Considering the weight of 0.12 kg and a 4800 times of use results in 576 kg of incinerated plastics
   1. Incineration of WIVA containers: Considering the weight of 1.08 kg and a 320 times of use results in 3345.6 kg of incinerated plastics. This results in a total of 921.6 kg.

### Supplementary Table S25 20 Liter scenario

| **20 liter scenario** | | | |
| --- | --- | --- | --- |
| **Economic inputs** | | | |
| **Product** | | **Amount** | **ecoinvent name** |
| 1 | Reusable cannister | 10 units | N/A |
| 2 | Single use cannister | 4000 units | N/A |
| 3 | WIVA container | 266 | N/A |
| 4 | Electricity use | 43.56 kWh | electricity, low voltage_market for electricity, low voltage[NL] |
| **Environmental inputs** | | | |
| **Product** | | **Amount** | **Ecoinvent name** |
|  | N/A |  |  |
| **Economic outputs** | | | |
| **Product** | | **Amount** | **Ecoinvent name** |
| 5 | Incinerated intra-operative collected fluids | 8000 liter | N/A |
| 6 | 1 year of use |  |  |
| 7 | Incineration of plastic | 767.28 kg | waste plastic, mixture_treatment of waste plastic, mixture, municipal incineration[RoW] |
| **Environmental outputs** | | | |
| **Product** | | **Amount** | **ecoinvent name** |
|  | N/A |  |  |

1. 10 per procedure as the reusable cannisters have a volume of 2 liter.
2. 10 per procedure with a volume of 2 liter. Hence, 4000 single use cannisters
3. Assumed that 0.33 container is required per procedure by dividing the volume with the liquids (20/30). Thus, a total of 166.66 containers.
4. Based on data sheet of fluid management system of Serres (n.d.) that uses a separate machine for fluid management with the use of cannisters (6x6L). This machine uses 330 VA, which equals 0.33 kW. With a duration 2 hours of which the machine is effectively used for 20 minutes, the total energy consumption is: 0.33*0.33*400 = 43.56 kWh.
5. The intra-operative collected fluids are directly incinerated together with the cannisters and WIVA container. In a year, 400 procedures are performed with an average of 10 liters. Thus, 10,000 liter is incinerated.
6. n/a
7. Incineration of single-use cannister: Considering the weight of 0.12 kg and a 4000 times of use results in 480 kg of incinerated plastics
8. Incineration of WIVA containers: Considering the weight of 1.08 kg and a 266 times of use results in 287.28 kg of incinerated plastics. This results in a total of 767.28 kg.

### Supplementary Table S26 10 Liter scenario

| **10 liter scenario** | | | |
| --- | --- | --- | --- |
| **Economic inputs** | | | |
| **Product** | | **Amount** | **Ecoinvent name** |
| 1 | Reusable cannister | 5 units | N/A |
| 2 | Single use cannister | 2000 units | N/A |
| 3 | WIVA container | 133 | N/A |
| 4 | Electricity use | 43.56 kWh | electricity, low voltage_market for electricity, low voltage[NL] |
| **Environmental inputs** | | | |
| **Product** | | **Amount** | **Ecoinvent name** |
|  | N/A |  |  |
| **Economic outputs** | | | |
| **Product** | | **Amount** | **Ecoinvent name** |
| 5 | Incinerated intra-operative collected fluids | 4000 liter | N/A |
| 6 | 1 year of use |  |  |
| 7 | Incineration of plastic | 383.64 kg | waste plastic, mixture_treatment of waste plastic, mixture, municipal incineration[RoW] |
| **Environmental outputs** | | | |
| **Product** | | **Amount** | **Ecoinvent name** |
|  | N/A |  |  |

1. 5 per procedure as the reusable cannisters have a volume of 2 liter.
2. 5 per procedure with a volume of 2 liter. Hence, 2000 single use cannisters
3. Assumed that 0.33 container is required per procedure by dividing the volume with the liquids (10/30). Thus, a total of 133.33 containers.
4. Based on data sheet of fluid management system of Serres (n.d.) that uses a separate machine for fluid management with the use of cannisters (6x6L). This machine uses 330 VA, which equals 0.33 kW. With a duration 2 hours of which the machine is effectively used for 20 minutes, the total energy consumption is: 0.33*0.33*400 = 43.56 kWh.
5. The intra-operative collected fluids are directly incinerated together with the cannisters and WIVA container. In a year, 400 procedures are performed with an average of 10 liters. Thus, 4000 liter is incinerated.
6. n/a
7. Incineration of single-use cannister: Considering the weight of 0.12 kg and a 2000 times of use results in 240 kg of incinerated plastics

Incineration of WIVA containers: Considering the weight of 1.08 kg and a 133 times of use results in 143.64 kg of incinerated plastics. This results in a total of 383.64 kg.

### Supplementary Table S27 7 Liter scenario

| **7 liter scenario** | | | |
| --- | --- | --- | --- |
| **Economic inputs** | | | |
| **Product** | | **Amount** | **Ecoinvent name** |
| 1 | Reusable cannister | 2.5 units | N/A |
| 2 | Single use cannister | 2000 units | N/A |
| 3 | WIVA container | 133 units | N/A |
| 4 | Electricity use | 87.12 kWh | electricity, low voltage_market for electricity, low voltage[NL] |
| **Environmental inputs** | | | |
| **Product** | | **Amount** | **Ecoinvent name** |
|  | N/A |  |  |
| **Economic outputs** | | | |
| **Product** | | **Amount** | **Ecoinvent name** |
| 5 | Incinerated intra-operative collected fluids | 4000 liter |  |
| 6 | 1 year of use |  |  |
| 7 | Incineration of plastic | 383.64 kg | waste plastic, mixture_treatment of waste plastic, mixture, municipal incineration[RoW] |
| **Environmental outputs** | | | |
| **Product** | | **Amount** | **Ecoinvent name** |
|  | N/A |  |  |

1. 4 per procedure as the reusable cannisters have a volume of 2 liter.
2. 4 per procedure with a volume of 2 liter. Hence, 1600 single use cannisters
3. Assumed that 0.23 container is required per procedure by dividing the volume with the liquids (7/30). Thus, a total of 93.33 containers.
4. Based on data sheet of fluid management system of Serres (n.d.) that uses a separate machine for fluid management with the use of cannisters (6x6L). This machine uses 330 VA, which equals 0.33 kW. With a duration 2 hours of which the machine is effectively used for 20 minutes, the total energy consumption is: 0.33*0.33*400 = 43.56 kWh.
5. The intra-operative collected fluids are directly incinerated together with the cannisters and WIVA container. In a year, 400 procedures are performed with an average of 7 liters. Thus, 2800 liter is incinerated.
6. n/a
7. Incineration of single-use cannister: Considering the weight of 0.12 kg and a 1600 times of use results in 192 kg of incinerated plastics

Incineration of WIVA containers: Considering the weight of 1.08 kg and a 93 times of use results in 100.80 kg of incinerated plastics. This results in a total of 292.80 kg.

### Supplementary Table S28 5 Liter scenario

| **5 liter scenario** | | | |
| --- | --- | --- | --- |
| **Economic inputs** | | | |
| **Product** | | **Amount** | **Ecoinvent name** |
| 1 | Reusable cannister | 4 units | N/A |
| 2 | Single use cannister | 1600 units | N/A |
| 3 | WIVA container | 93.33 units | N/A |
| 4 | Electricity use | 43.56 kWh | electricity, low voltage_market for electricity, low voltage[NL] |
| **Environmental inputs** | | | |
| **Product** | | **Amount** | **Ecoinvent name** |
|  | N/A |  |  |
| **Economic outputs** | | | |
| **Product** | | **Amount** | **Ecoinvent name** |
| 5 | Incinerated intra-operative collected fluids | 2800 liter |  |
| 6 | 1 year of use |  |  |
| 7 | Incineration of plastic | 292.80 kg | waste plastic, mixture_treatment of waste plastic, mixture, municipal incineration[RoW] |
| **Environmental outputs** | | | |
| **Product** | | **Amount** | **Ecoinvent name** |
|  | N/A |  |  |

1. 2.5 per procedure as the reusable cannisters have a volume of 2 liter.
2. 2.5 per procedure with a volume of 2 liter. Hence, 2000 single use cannisters
3. Assumed that 0.17 container is required per procedure by dividing the volume with the liquids (5/30). Thus, a total of 133 containers.
4. Based on data sheet of fluid management system of Serres (n.d.) that uses a separate machine for fluid management with the use of cannisters (6x6L). This machine uses 330 VA, which equals 0.33 kW. With a duration 2 hours of which the machine is effectively used for 20 minutes, the total energy consumption is: 0.33*0.33*800 = 87.12 kWh.
5. The intra-operative collected fluids are directly incinerated together with the cannisters and WIVA container. In a year, 800 procedures are performed with an average of 5 liters. Thus, 4000 liter is incinerated.
6. n/a
7. Incineration of single-use cannister: Considering the weight of 0.12 kg and a 2000 times of use results in 240 kg of incinerated plastics

Incineration of WIVA containers: Considering the weight of 1.08 kg and a 133 times of use results in 143.64 kg of incinerated plastics. This results in a total of 383.64 kg.

Supplementary Table S29 2 Liter scenario

| **2 liter scenario** | | | |
| --- | --- | --- | --- |
| **Economic inputs** | | | |
| **Product** | | **Amount** | **Ecoinvent name** |
| 1 | Reusable cannister | 1 units | N/A |
| 2 | Single use cannister | 800 units | N/A |
| 3 | WIVA container | 56 units | N/A |
| 4 | Electricity use | 87.12 kWh | electricity, low voltage_market for electricity, low voltage[NL] |
| **Environmental inputs** | | | |
| **Product** | | **Amount** | **Ecoinvent name** |
|  | N/A |  |  |
| **Economic outputs** | | | |
| **Product** | | **Amount** | **Ecoinvent name** |
| 5 | Incinerated intra-operative collected fluids | 1600 liter |  |
| 6 | 1 year of use |  |  |
| 7 | Incineration of plastic | 153.24 | waste plastic, mixture_treatment of waste plastic, mixture, municipal incineration[RoW] |
| **Environmental outputs** | | | |
| **Product** | | **Amount** | **Ecoinvent name** |
|  | N/A |  |  |

1. 1 per procedure as the reusable cannisters have a volume of 2 liter.
2. 1 per procedure with a volume of 2 liter. Hence, 800 single use cannisters
3. Assumed that 0.07 container is required per procedure by dividing the volume with the liquids (2/30). Thus, a total of 56 containers.
4. Based on data sheet of fluid management system of Serres (n.d.) that uses a separate machine for fluid management with the use of cannisters (6x6L). This machine uses 330 VA, which equals 0.33 kW. With a duration 2 hours of which the machine is effectively used for 20 minutes, the total energy consumption is: 0.33*0.33*800 = 87.12 kWh.
5. The intra-operative collected fluids are directly incinerated together with the cannisters and WIVA container. In a year, 800 procedures are performed with an average of 2 liters. Thus, 1600 liter is incinerated.
6. n/a
7. Incineration of single-use cannister: Considering the weight of 0.12 kg and a 800 times of use results in 96 kg of incinerated plastics

Incineration of WIVA containers: Considering the weight of 1.08 kg and a 53 times of use results in 57.24 kg of incinerated plastics. This results in a total of 153.24 kg.

### Supplementary Table S30 500 cc scenario

| **500CC scenario** | | | |
| --- | --- | --- | --- |
| **Economic inputs** | | | |
| **Product** | | **Amount** | **Ecoinvent name** |
| 1 | Reusable cannister | 1 units | N/A |
| 2 | Single use cannister | 550 units | N/A |
| 3 | WIVA container | 36.67 units | N/A |
| 4 | Electricity use | 59.90 kWh | electricity, low voltage_market for electricity, low voltage[NL] |
| **Environmental inputs** | | | |
| **Product** | | **Amount** | **Ecoinvent name** |
|  | N/A |  |  |
| **Economic outputs** | | | |
| **Product** | | **Amount** | **Ecoinvent name** |
| 5 | Incinerated intra-operative collected fluids | 275 liter |  |
| 6 | 1 year of use |  |  |
| 7 | Incineration of plastic | 105.60 | waste plastic, mixture_treatment of waste plastic, mixture, municipal incineration[RoW] |
| **Environmental outputs** | | | |
| **Product** | | **Amount** | **Ecoinvent name** |
|  | N/A |  |  |

1. 1 per procedure as the reusable cannisters have a volume of 2 liter.
2. 1 per procedure with a volume of 2 liter. Hence, 550 single use cannisters
3. Assumed that 0.06 container is required per procedure by dividing the volume with the wiva container (2/30). Thus, a total of 36.67 containers.
4. Based on data sheet of fluid management system of Serres (n.d.) that uses a separate machine for fluid management with the use of cannisters (6x6L). This machine uses 330 VA, which equals 0.33 kW. With a duration 2 hours of which the machine is effectively used for 20 minutes, the total energy consumption is: 0.33*0.33*550 = 59.90 kWh.
5. The intra-operative collected fluids are directly incinerated together with the cannisters and WIVA container. In a year, 550 procedures are performed with an average of 0.5 liters. Thus, 275 liter is incinerated.
6. n/a
7. Incineration of single-use cannister: Considering the weight of 0.12 kg and a 550 times of use results in 66 kg of incinerated plastics

Incineration of WIVA containers: Considering the weight of 1.08 kg and a 36.67 times of use results in 39.60 kg of incinerated plastics. This results in a total of 105.60 kg.

### Supplementary Table S31 400 cc scenario

| **400CC scenario** | | | |
| --- | --- | --- | --- |
| **Economic inputs** | | | |
| **Product** | | **Amount** | **Ecoinvent name** |
| 1 | Reusable cannister | 1 units | N/A |
| 2 | Single use cannister | 550 units | N/A |
| 3 | WIVA container | 36.67 units | N/A |
| 4 | Electricity use | 59.90 kWh | electricity, low voltage_market for electricity, low voltage[NL] |
| **Environmental inputs** | | | |
| **Product** | | **Amount** | **Ecoinvent name** |
|  | N/A |  |  |
| **Economic outputs** | | | |
| **Product** | | **Amount** | **Ecoinvent name** |
| 5 | Incinerated intra-operative collected fluids | 220 liter |  |
| 6 | 1 year of use |  |  |
| 7 | Incineration of plastic | 105.60 | waste plastic, mixture_treatment of waste plastic, mixture, municipal incineration[RoW] |
| **Environmental outputs** | | | |
| **Product** | | **Amount** | **Ecoinvent name** |
|  | N/A |  |  |

1. 1 per procedure as the reusable cannisters have a volume of 2 liter.
2. 1 per procedure with a volume of 2 liter. Hence, 550 single use cannisters
3. Assumed that 0.06 container is required per procedure by dividing the volume with the wiva container (2/30). Thus, a total of 36.67containers.
4. Based on data sheet of fluid management system of Serres (n.d.) that uses a separate machine for fluid management with the use of cannisters (6x6L). This machine uses 330 VA, which equals 0.33 kW. With a duration 2 hours of which the machine is effectively used for 20 minutes, the total energy consumption is: 0.33*0.33*550 = 59.90 kWh.
5. The intra-operative collected fluids are directly incinerated together with the cannisters and WIVA container. In a year, 550 procedures are performed with an average of 0.4 liters. Thus, 220 liter is incinerated.
6. n/a
7. Incineration of single-use cannister: Considering the weight of 0.12 kg and a 550 times of use results in 66 kg of incinerated plastics

Incineration of WIVA containers: Considering the weight of 1.08 kg and a 36.67 times of use results in 39.60 kg of incinerated plastics. This results in a total of 105.60 kg.

### Supplementary Table S32 300 cc scenario

| **300CC scenario** | | | |
| --- | --- | --- | --- |
| **Economic inputs** | | | |
| **Product** | | **Amount** | **Ecoinvent name** |
| 1 | Reusable cannister | 1 units | N/A |
| 2 | Single use cannister | 550 units | N/A |
| 3 | WIVA container | 36.67 units | N/A |
| 4 | Electricity use | 59.90 kWh | electricity, low voltage_market for electricity, low voltage[NL] |
| **Environmental inputs** | | | |
| **Product** | | **Amount** | **Ecoinvent name** |
|  | N/A |  |  |
| **Economic outputs** | | | |
| **Product** | | **Amount** | **Ecoinvent name** |
| 5 | Incinerated intra-operative collected fluids | 165 liter |  |
| 6 | 1 year of use |  |  |
| 7 | Incineration of plastic | 153.24 | waste plastic, mixture_treatment of waste plastic, mixture, municipal incineration[RoW] |
| **Environmental outputs** | | | |
| **Product** | | **Amount** | **Ecoinvent name** |
|  | N/A |  |  |

1. 1 per procedure as the reusable cannisters have a volume of 2 liter.
2. 1 per procedure with a volume of 2 liter. Hence, 550 single use cannisters
3. Assumed that 0.06 container is required per procedure by dividing the volume with the liquids (02/30). Thus, a total of 36.67 containers.
4. Based on data sheet of fluid management system of Serres (n.d.) that uses a separate machine for fluid management with the use of cannisters (6x6L). This machine uses 330 VA, which equals 0.33 kW. With a duration 2 hours of which the machine is effectively used for 20 minutes, the total energy consumption is: 0.33*0.33*550 = 59.90 kWh.
5. The intra-operative collected fluids are directly incinerated together with the cannisters and WIVA container. In a year, 550 procedures are performed with an average of 0.3 liters. Thus, 165 liter is incinerated.
6. n/a
7. Incineration of single-use cannister: Considering the weight of 0.12 kg and a 550 times of use results in 66 kg of incinerated plastics

Incineration of WIVA containers: Considering the weight of 1.08 kg and a 36.67 times of use results in 39.60 kg of incinerated plastics. This results in a total of 105.60 kg.

### Supplementary Table S33 200 cc scenario

| **200CC scenario** | | | |
| --- | --- | --- | --- |
| **Economic inputs** | | | |
| **Product** | | **Amount** | **Ecoinvent name** |
| 1 | Reusable cannister | 1 units | N/A |
| 2 | Single use cannister | 550 units | N/A |
| 3 | WIVA container | 36.67 units | N/A |
| 4 | Electricity use | 59.90 kWh | electricity, low voltage_market for electricity, low voltage[NL] |
| **Environmental inputs** | | | |
| **Product** | | **Amount** | **Ecoinvent name** |
|  | N/A |  |  |
| **Economic outputs** | | | |
| **Product** | | **Amount** | **Ecoinvent name** |
| 5 | Incinerated intra-operative collected fluids | 110 liter |  |
| 6 | 1 year of use |  |  |
| 7 | Incineration of plastic | 153.24 | waste plastic, mixture_treatment of waste plastic, mixture, municipal incineration[RoW] |
| **Environmental outputs** | | | |
| **Product** | | **Amount** | **Ecoinvent name** |
|  | N/A |  |  |

1. 1 per procedure as the reusable cannisters have a volume of 2 liter.
2. 1 per procedure with a volume of 2 liter. Hence, 550 single use cannisters
3. Assumed that 0.06 container is required per procedure by dividing the volume with the liquids (02/30). Thus, a total of 36.67 containers.
4. Based on data sheet of fluid management system of Serres (n.d.) that uses a separate machine for fluid management with the use of cannisters (6x6L). This machine uses 330 VA, which equals 0.33 kW. With a duration 2 hours of which the machine is effectively used for 20 minutes, the total energy consumption is: 0.33*0.33*550 = 59.90 kWh.
5. The intra-operative collected fluids are directly incinerated together with the cannisters and WIVA container. In a year, 550 procedures are performed with an average of 0.2 liters. Thus, 110 liter is incinerated.
6. n/a
7. Incineration of single-use cannister: Considering the weight of 0.12 kg and a 550 times of use results in 66 kg of incinerated plastics

Incineration of WIVA containers: Considering the weight of 1.08 kg and a 36.67 times of use results in 39.60 kg of incinerated plastics. This results in a total of 105.60 kg.

### Supplementary Table S34 100 cc scenario

| **100CC scenario** | | | |
| --- | --- | --- | --- |
| **Economic inputs** | | | |
| **Product** | | **Amount** | **Ecoinvent name** |
| 1 | Reusable cannister | 1 units | N/A |
| 2 | Single use cannister | 550 units | N/A |
| 3 | WIVA container | 36.67 units | N/A |
| 4 | Electricity use | 59.90 kWh | electricity, low voltage_market for electricity, low voltage[NL] |
| **Environmental inputs** | | | |
| **Product** | | **Amount** | **Ecoinvent name** |
|  | N/A |  |  |
| **Economic outputs** | | | |
| **Product** | | **Amount** | **Ecoinvent name** |
| 5 | Incinerated intra-operative collected fluids | 55 liter |  |
| 6 | 1 year of use |  |  |
| 7 | Incineration of plastic | 153.24 | waste plastic, mixture_treatment of waste plastic, mixture, municipal incineration[RoW] |
| **Environmental outputs** | | | |
| **Product** | | **Amount** | **Ecoinvent name** |
|  | N/A |  |  |

1. 1 per procedure as the reusable cannisters have a volume of 2 liter.
2. 1 per procedure with a volume of 2 liter. Hence, 550 single use cannisters
3. Assumed that 0.06 container is required per procedure by dividing the volume with the liquids (02/30). Thus, a total of 36.67 containers.
4. Based on data sheet of fluid management system of Serres (n.d.) that uses a separate machine for fluid management with the use of cannisters (6x6L). This machine uses 330 VA, which equals 0.33 kW. With a duration 2 hours of which the machine is effectively used for 20 minutes, the total energy consumption is: 0.33*0.33*550 = 59.90 kWh.
5. The intra-operative collected fluids are directly incinerated together with the cannisters and WIVA container. In a year, 550 procedures are performed with an average of 012 liters. Thus, 55 liter is incinerated.
6. n/a
7. Incineration of single-use cannister: Considering the weight of 0.12 kg and a 550 times of use results in 66 kg of incinerated plastics

Incineration of WIVA containers: Considering the weight of 1.08 kg and a 36.67 times of use results in 39.60 kg of incinerated plastics. This results in a total of 105.60 kg.

## Disposal

The inventory data used to model the treatment of plastic waste from the use of the cannisters and WIVA containers is obtained from Ecoinvent.

## Incineration of liquids

### Supplementary Table S35 Incineration of liquids

| **Incineration of liquids** | | | |
| --- | --- | --- | --- |
| **Economic inputs** | | | |
| **Product** | | **Amount** | **Ecoinvent name** |
| 1 | Road freight | 0.0911 tkm | transport, freight, lorry 16-32 metric ton, EURO6_transport, freight, lorry 16-32 metric ton, EURO6[RoW] |
| 2 | heat | 0.36 MJ | heat, district or industrial, natural gas_heat and power co-generation, natural gas, conventional power plant, 100MW electrical[NL] |
| 3 | Municipal waste incineration facility | 2.5E-10 unit | municipal waste incineration facility_market for municipal waste incineration facility[RoW] |
| 4 | intra-operative collected fluids | 1 liter | N/A |
| **Environmental inputs** | | | |
| **Product** | | **Amount** | **Ecoinvent name** |
|  | N/A |  |  |
| **Economic outputs** | | | |
| **Product** | | **Amount** | **Ecoinvent name** |
| 4 | Incinerated intra-operative collected fluids | 1 liter |  |
| **Environmental outputs** | | | |
| **Product** | | **Amount** | **Ecoinvent name** |
| 5 | Heat | 0.36 MJ | Heat, waste[('air', 'non-urban air or from high stacks')] |
| 6 | Water | 0.001 m3 | Water[('air', 'non-urban air or from high stacks')] |

1. The assumed distance between the hospital and the special waste incineration site is assumed to be 91.1 km and a total of 0.001 ton of liquids are transported.
2. The required heat to evaporate 1 kg of water is calculated as following:

$$Q= m \cdot c \cdot\Delta T$$

Q = heat energy [J] = 0.36 MJ

m = mass [kg] = 1kg

c = specific heat [J/kg∙ °C] = 4186 J/kg∙ °C

∆T = difference in temperature [°C] = 100 – 15 = 85 °C

1. Based on incineration process obtained from Ecoinvent
2. N/A
3. Assumed that the additional heat to evaporate the water is leaving the economic system.
4. Assumed that the evaporated water is leaving the economic water as steam.

### Energy

The inventory data used to model the energy that is used to operate the Neptune and docking station is obtained from Ecoinvent.
